# Application of an improved YOLOv5s-based deep learning model for automated detection of pulmonary adenocarcinoma *in situ* and minimally invasive adenocarcinoma

**DOI:** 10.3389/fmed.2026.1885660

**Published:** 2026-07-24

**Authors:** Zhipeng Sun, Jinghui Chen, Lianxin Xie, Tao Yang, Lanlan Yang, Chengbin Ye, Hongjia Zhao

**Affiliations:** 1 The First Clinical Medical College, The Affiliated People's Hospital of Fujian University of Traditional Chinese Medicine, Fuzhou, Fujian, China; 2 Department of Medical Imaging, Chongqing Eye and Optometry Ophthalmology Hospital, Chongqing, China; 3 Department of Medical Imaging, The Affiliated People's Hospital of Fujian University of Traditional Chinese Medicine, Fuzhou, Fujian, China; 4 The Affiliated People's Hospital of Fujian University of Traditional Chinese Medicine, Fuzhou, Fujian, China

**Keywords:** attention mechanism, deep learning, minimally invasive adenocarcinoma, pulmonary adenocarcinoma *in situ*, YOLOv5S

## Abstract

**Objective:**

To investigate the performance and clinical application potential of an improved YOLOv5-based deep learning model for automated detection and classification of pulmonary adenocarcinoma *in situ* (AIS) and minimally invasive adenocarcinoma (MIA), with particular emphasis on attention mechanisms and multiscale feature fusion for small pulmonary nodule detection.

**Methods:**

CT images of 225 pathologically confirmed AIS or MIA lesions treated at the Affiliated People's Hospital of Fujian University of Traditional Chinese Medicine between December 2019 and June 2025 were retrospectively collected. In the deep learning workflow, the 225 lesions were first divided at the lesion level: 27 lesions (11 MIA and 16 AIS) were set aside, and the 218 original PNG images derived from these lesions were reserved as an independent test set that was excluded from all data augmentation and model training. The remaining lesions were converted into PNG format and further augmented using horizontal flipping, yielding a final dataset of 3,000 images for training and validation. Subsequently, the dataset was partitioned into training and validation sets at an 8:2 ratio. During this process, the origins of the augmented lesion images were manually verified to ensure that an original image and its corresponding augmented variants were not assigned to different subsets simultaneously. YOLOv5s was used as the baseline model. Local contrast-spatial attention modules (LCSA and LCSAv2), BiFPN, and ASFF were incorporated for attention enhancement and multiscale feature fusion. Five-fold cross-validation was performed. Mean average precision (mAP), precision, and recall were evaluated across IoU thresholds from 0.6 to 0.9, and ablation experiments were conducted to quantify the contribution of each module.

**Results:**

In the baseline model selection stage, comparison under identical conditions showed that YOLOv5s achieved detection performance comparable to YOLOv11s while reducing GFLOPs by approximately 25%; therefore, YOLOv5s was selected as the improvement baseline. In five-fold cross-validation at IoU = 0.6, baseline YOLOv5s achieved an mAP@50 (AP at the matching IoU of 0.6) of 0.947, precision of 0.849, and recall of 0.932. After LCSA x ASFF was introduced, recall increased to 0.941 and mAP@50 was maintained at 0.946. Under the stringent localization criterion of IoU = 0.9, LCSA × ASFF achieved an mAP@50 of 0.878, a 3.8-percentage-point higher value than the baseline (0.846); precision (0.879) and recall (0.773) were also the highest among all models, with the smallest performance attenuation in the high-IoU range. Ablation experiments showed that the combined use of LCSA and ASFF produced stronger synergistic effects in the high-IoU range than either module alone, whereas LCSAv2 combined with ASFF did not show the expected synergistic gain. On the independent test set, baseline YOLOv5s achieved precision, recall, and mAP@50 values of 0.747, 0.715, and 0.753, respectively; the improved LCSA × ASFF model achieved corresponding values of 0.693, 0.753, and 0.788. Recall and mAP@50 increased simultaneously, indicating better overall detection performance than the baseline. The improved YOLOv5s-based deep learning model performed better in automated detection, and the LCSA × ASFF combination improved localization accuracy while maintaining recall through the synergy between local contrast enhancement and adaptive scale fusion.

**Conclusion:**

The improved YOLOv5s model achieved automated detection and classification of small pulmonary nodules through end-to-end training, showing clear advantages in clinical workflow automation without manual intervention. The synergistic effect of local contrast enhancement and adaptive scale fusion effectively improved AIS/MIA localization precision and classification accuracy, supporting its potential as an assistive tool for early lung adenocarcinoma screening, pending external multi-centre validation.

## 1 Introduction

Lung cancer, particularly lung adenocarcinoma, is one of the leading causes of cancer-related death worldwide. Data from 2020 showed approximately 2.2 million new lung cancer cases and approximately 1.8 million deaths worldwide (1). Adenocarcinoma is the most common histological type of lung cancer, and its incidence continues to increase. Pulmonary adenocarcinoma *in situ* (AIS) and minimally invasive adenocarcinoma (MIA) are important pathological subtypes of early lung adenocarcinoma. According to the 2021 World Health Organization histological classification of lung tumors (2), AIS is classified as a glandular precursor lesion, namely a precancerous lesion. For AIS, treatment can be determined through dynamic observation and follow-up. If a glandular precursor lesion shows signs of progression toward minimally invasive or invasive lung adenocarcinoma, timely surgical resection is required (3).

On conventional computed tomography (CT), AIS and MIA often present as ground-glass nodules (GGNs) (4). AIS usually appear as a pure ground-glass opacity (GGO) nodule with a clear or slightly irregular margin and without an obvious internal solid component, which is consistent with its non-invasive pathological features. MIA is mainly characterized by ground-glass opacity with a focal solid component (5); pathologically, the solid region corresponds to a microinvasive focus and suggests potential invasive behavior. Multiple studies have shown that the solid-component size and CT attenuation of MIA are usually higher than those of AIS, and the margin may show spiculation or lobulation, with subtle local traction of pulmonary markings or an air bronchogram sign (6). Texture-level differences are also documented: gray-level contrast, texture complexity, and internal heterogeneity are significantly higher in MIA nodules than in AIS nodules (7), providing an imaging basis for quantitative discrimination between the two lesion types.

However, nodule location, volume differences, scanning parameters, and other factors can interfere with imaging appearance, thereby limiting the accuracy of conventional visual CT interpretation. Reliance on morphology alone is insufficient for consistently distinguishing AIS from MIA. Quantitative imaging methods or computer-aided diagnostic models are therefore needed to improve non-invasive recognition (8), with practical clinical value for surgical decision-making and follow-up planning.

As the application of artificial intelligence (AI) in the field of medical imaging continues to deepen, its role has gradually transformed from an early auxiliary tool to an important part of the clinical decision support system (9). Traditional imaging diagnosis relies on the experience accumulation and visual judgment of radiologists. When faced with early-stage lung adenocarcinoma lesions with subtle and highly overlapping imaging features such as AIS and MIA, the limitations of subjective interpretation are particularly prominent. It is difficult to achieve high sensitivity and high specificity simultaneously (10). The introduction of AI technology has opened up a new technical path to improve the objectivity, accuracy, and automation level of image analysis.

The deep learning end-to-end model automatically extracts image representations through convolutional neural networks without explicit feature engineering intervention. Its excellent performance in image classification and object detection tasks makes it an important technical support for automatic detection and differential diagnosis of pulmonary nodules (11). The YOLO (You Only Look Once) series of object detection networks use a unified end-to-end framework to achieve fast and accurate small object localization, providing a feasible methodological basis for the automatic identification of tiny lesions (12).

Deep learning object detection methods are usually divided into two types of frameworks: two-stage and single-stage frameworks. The two-stage detection is represented by the R-CNN series. The Region Proposal Network (RPN) first generates candidate boxes, and then performs classification and regression optimization on the candidate areas. The stability of small object detection is relatively high. Setio et al. (13) proposed a multi-view CNN detection system based on the LUNA16 dataset, with an average recall rate of 85.4% in public benchmark testing; the three-dimensional deep convolutional network built by Hamidian et al. (14) reported a sensitivity of more than 80% on the same dataset. The single-stage detection method uses a single forward propagation to complete target location and classification simultaneously, and the inference efficiency is significantly higher than that of the two-stage framework. In recent years, many studies have verified improved models such as YOLOv5 on LUNA16 and clinical CT data. The results show that this type of method can achieve a good balance between speed and accuracy, and has the potential for clinical real-time application.

For the classification of lung cancer, Zhao et al. established a deep learning model based on a 3D convolutional neural network to predict the invasiveness of lung adenocarcinoma. In multi-category tasks including atypical adenomatous hyperplasia, AIS, MIA, and IAC, the weighted F1 value of their model was 0.633, and the overall performance was better than that of many radiologists (15). Wang et al. (16) proposed a model framework that combines segmentation and classification to achieve better AUC performance than traditional machine learning methods in the task of pathological classification of ground-glass nodules. Qi et al. (17) proposed the Lung-PNet model for automatic segmentation and invasiveness prediction of pure ground-glass nodules, which showed good discriminant performance in multi-center validation. Building on these efforts, recent convolutional pipelines have further advanced automated lung-lesion detection and segmentation in chest CT, including segmentation-enhanced CNN-ensemble methods and U-Net architectures with CNN backbones (18, 19).

Among these detection frameworks, the single-stage detection model has attracted attention due to its end-to-end training and efficient inference properties, especially suitable for rapid detection and classification of tiny nodules. Against this background, the YOLO series models have been widely used in the fields of conventional images and medical imaging due to their efficient inference and powerful multi-scale detection capabilities. YOLO was proposed by Redmon et al. (20), is a representative framework of single-stage object detection algorithm. Its core design converts the object detection problem into a single forward propagation regression task. The input image is divided into S × S grids. Each grid directly predicts a fixed number of bounding box coordinates, confidence, and category probabilities. The positioning error, confidence error, and classification error are simultaneously optimized through a joint loss function. Compared with two-stage methods such as R-CNN, which require independent candidate region generation and repeated feature extraction, YOLO's end-to-end training achieves whole-image feature sharing, and the inference speed is greatly improved, reaching 45 fps on the PASCAL VOC 2007 dataset, while maintaining competitive mAP performance.

Since then, the YOLO series has experienced continuous structural iterations. YOLOv2 and YOLOv3 successively introduced the anchor mechanism, multi-scale prediction structure and deeper network design, and the detection accuracy and small target recognition capabilities were significantly improved (21).

On this basis, YOLOv5 integrates the cross-stage partial connection structure, adaptive anchor frame mechanism and more efficient feature pyramid design, further balancing real-time performance and detection performance. According to the model scale, YOLOv5 is divided into different variants such as s, m, l, x, etc. to adapt to differentiated computing resource requirements and deployment scenarios.

YOLOv5s is the variant with the smallest number of parameters and the fastest inference speed in the YOLOv5 series. Maintaining strong feature representation and multi-scale detection capabilities under lightweight constraints is its core design goal. Different from the two-stage detection framework that relies on candidate region generation, YOLOv5s adopts a unified end-to-end regression strategy. Object localization and classification are collaboratively modeled in a single forward propagation, thus greatly improving the inference efficiency and real-time detection capabilities (22).

Backbone uses a cross-stage partial connection structure variant as the backbone network to shunt feature maps, effectively reducing repeated calculations of gradients while retaining deep semantic information. It has potential advantages for learning edge features of low-contrast small targets (23). The Neck part introduces the PANet path aggregation network. The top-down and bottom-up bidirectional feature flow fully integrates deep semantics and shallow detail information at multiple levels, and the detection ability of small-scale targets is thus strengthened (24).

YOLOv5s does not rely on complex attention modules or huge network stacks. Instead, it achieves end-to-end optimization through efficient feature fusion paths and anchor frame mechanisms. It achieves high mAP and inference speeds under the premise of controlled parameter scale, and is suitable for lightweight deployment of real-time diagnostic assistance systems (25).

The small object detection rate is a long-standing performance bottleneck in deep learning applications in medical imaging. AIS and MIA are mostly presented in the form of GGN. On CT images, they appear to have low density, blurred boundaries, and little contrast with surrounding normal lung tissue, which poses great challenges to the effective detection of deep networks (26). Compared with large-sized solid lesions, AIS and MIA occupy a very small space. The blurred gray edges make it difficult for shallow convolution to extract stable local discriminant signals. The risk of information loss in deep feature maps further intensifies as the downsampling multiple increases (27).

Faced with this problem, multi-scale information fusion is crucial to improving detection performance. Although single-stage detection frameworks such as YOLOv5s achieve cross-scale feature integration through FPN and PAN, there are still shortcomings in the effective utilization of extremely small targets in high-resolution medical images (24). Enhanced feature fusion modules such as Bi-directional Feature Pyramid Network (BiFPN) and Adaptive Spatial Feature Fusion (ASFF) have been proposed to improve scale adaptability. However, balancing computational overhead and feature representation capabilities while maintaining lightweight deployment conditions is still a common challenge faced by such methods (28). The problem of class imbalance cannot be ignored. There are far more background areas than nodule areas in lung nodule data. Samples of pathological subtypes such as AIS/MIA are relatively scarce. During the model training process, prediction shifts to background or high-frequency categories are prone to occur. Although focus loss or resampling strategies can alleviate this problem to a certain extent, it is difficult to fundamentally eliminate the impact of imbalanced class distribution on discriminative ability (29).

Even when detection and classification are achieved, localization accuracy remains a major challenge. During bounding-box regression, the smaller the target, the more sensitive the Intersection over Union (IoU) loss is to small deviations in the predicted box. Error accumulation can directly reduce detection accuracy and subsequent classification performance. The AIS/MIA task is particularly sensitive to this issue because subtle changes in the solid component within a nodule may correspond to different pathological classifications. Therefore, the model must retain sufficient spatial resolution during localization; otherwise, boundary-box deviation may lead to inaccurate pathological prediction.

Taken together, these observations indicate that, in AIS/MIA classification, deep convolutional networks are affected not only by information loss after downsampling but also by weak feature representation and background interference.

Attention mechanisms address this need. The attention mechanism is derived from the selective attention principle of human vision. Its core is to enhance the network's response ability to key areas by giving higher weight to important information in input features while suppressing irrelevant features (30). In convolutional neural networks, the attention mechanism is mainly divided into channel attention and spatial attention. Channel attention enhances key feature dimensions by calculating the importance coefficient of each feature channel; spatial attention generates a spatial attention map to highlight the significance of the target area and focus the network on the target location. The combination of channels and spatial attention can simultaneously strengthen the expression of information in feature dimensions and spatial positions, and is especially suitable for the detection and classification of small lesions.

In the study of attention mechanisms, the SE module proposed by Hu et al. (31) realizes feature recalibration through channel attention, significantly improving feature representation capabilities; Roy et al. (32) introduced spatial attention gating in Attention U-Net, which effectively enhanced the response of the target area and showed performance improvement in a variety of medical image segmentation tasks. In the field of pulmonary nodule detection, Cao et al. (33) constructed a multi-scale attention fusion network and improved detection sensitivity on the LUNA16 dataset; Li et al.'s (34) multi-scale detection system designed based on the global channel-spatial attention mechanism achieved a CPM value of 0.929 on LUNA16, further verifying the effectiveness of attention enhancement for small nodule detection. Beyond pulmonary nodules, attention-enhanced deep-learning architectures have similarly improved fine-grained classification in other radiological imaging tasks, such as the diagnosis of dental conditions from panoramic radiographs (35, 36), supporting the design of a task-specific attention module rather than a generic transplant of existing mechanisms for the AIS/MIA task.

Attention mechanism and multiscale feature fusion are naturally complementary at the mechanism level. The former implements directional enhancement of the characteristic responses of solid components and microinvasive structures inside nodules, while the latter alleviates the attenuation problem of small-object signals in deep networks. The two act on two independent dimensions: feature representation quality and cross-scale information utilization efficiency, respectively. Joint deployment is expected to improve the detection recall rate while providing higher-quality candidate region representations for pathological subtype classification. It is a feasible technical solution to improve the intelligent recognition performance of AIS and MIA.

**Figure 1:**
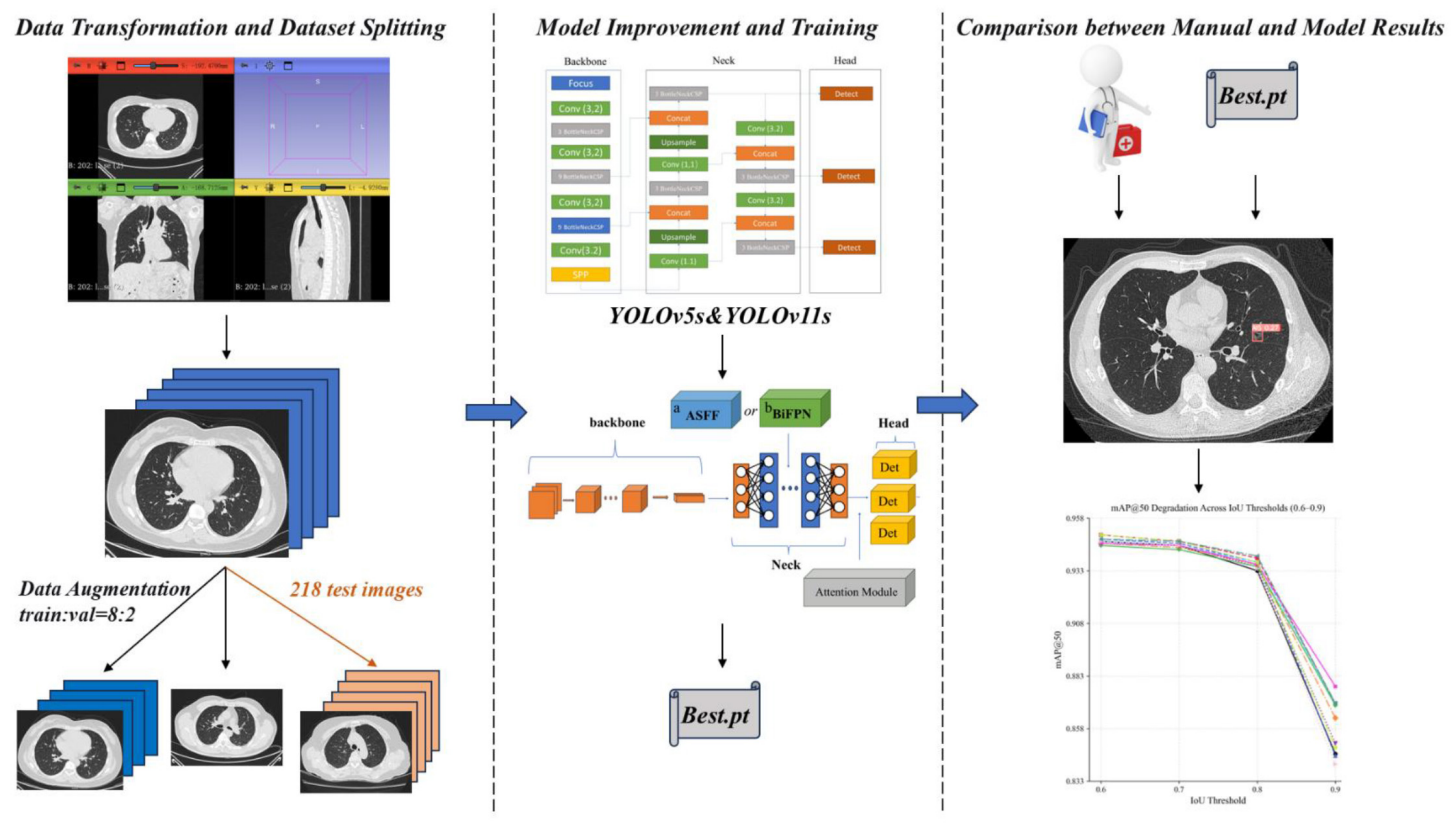
Technology roadmap of this research.

As shown in Figure 1, this study builds a deep learning detection model based on improved YOLOv5s. The CT imaging data of 225 lesions pathologically diagnosed as AIS or MIA collected by the Affiliated People's Hospital of Fujian University of Traditional Chinese Medicine were included.

Structural improvements were developed using the YOLOv5s object detection framework as the baseline model. The original DICOM format CT images were converted into PNG format in batches, and 218 images were first selected as a test set for generalization testing. The remaining samples were expanded to 3,000 images without changing the structural characteristics of the lesions, and LabelImg was used to complete the lesion boundary box annotation. The 3,000 images were divided into training sets and validation sets in a ratio of 8:2. Based on the baseline model, system optimization was carried out around feature representation and multi-scale fusion capabilities: BiFPN and ASFF were introduced to enhance bidirectional feature fusion and cross-scale adaptive alignment capabilities respectively; a local contrast spatial attention module [Local Contrast-Spatial Attention (LCSA)] was designed to enhance lesion boundary feature representation, and based on this, an improved version LCSAv2 was proposed to optimize the feature response mechanism. Ablation experiments are carried out through a variety of module combinations, and the independent contributions and synergistic effects of each module are systematically analyzed. In order to evaluate the competitiveness between YOLOv5s and newer architectures, this study conducted the same training comparison between YOLOv5s and the more recent YOLOv11s architecture under the same conditions, and then carried out systematic structural improvements around YOLOv5s. Indicators such as mAP, precision, and recall were calculated under different IoU thresholds (0.6–0.9), and the best structural model obtained was tested on an independent test set. Two doctors with more than 2 years of diagnostic experience were randomly invited to interpret the test set, and the results of manual interpretation compared with the model were analyzed.

## 2 Research objects and data sources

This retrospective study was approved by the Institutional Review Board of the Affiliated People's Hospital of Fujian University of Traditional Chinese Medicine (Approval No. YJS2025-147-02). We retrospectively collected chest computed tomography (CT) imaging data from patients who underwent diagnosis and treatment at this institution between December 2019 and June 2025. A total of 225 lesions, definitively diagnosed as adenocarcinoma *in situ* (AIS) or minimally invasive adenocarcinoma (MIA) via postoperative histopathological examination, were enrolled as the study dataset.

The inclusion criteria include: ① Receive chest CT examination within 1 month before surgery; ② The minimum diameter of the nodule is ≥2 mm and the maximum diameter is ≤ 3.0 cm; ③ Reconstruct thin-slice images, and the thickness of the chest CT image is 1 mm; ④ Diagnosed as AIS or MIA through pathological examination. Exclusion criteria include: ① image artifacts affecting diagnosis; ② history of other malignant tumors; ③ receipt of anti-tumor treatment before CT examination.

### 2.1 DICOM image conversion and unified processing

In this study, an improved YOLOv5s deep learning model was constructed using the CT images of 225 AIS and MIA lesions confirmed by postoperative pathology to achieve the automated localization and classification of small nodules.

Original medical images are usually stored in DICOM format, which not only contains pixel matrices, but also contains rich scanning parameters, such as layer thickness, reconstruction algorithm, window width and window level, scanning sequence information, etc. Although this information is of great significance for imaging diagnosis, in deep learning model training, the network mainly relies on standard image formats (such as PNG or JPEG) for input. Therefore, in order to ensure that the training data can be efficiently read by the model and retain the subtle structural characteristics of the lesions, when constructing a deep learning dataset, it is usually necessary to convert CT images in DICOM format into common formats such as PNG.

During the conversion process, all CT images are denoised and grayscale unified before processing to reduce the impact of different scanning equipment or parameters on image features. Subsequently, the processed image is converted to PNG format through a Python script. PNG adopts a lossless compression mechanism. The entire compression process does not introduce any image distortion or artifacts, and the grayscale levels and structural details are fully preserved. Compared with lossy compression formats, this conversion method can effectively avoid the risk of information attenuation and provide input data with stable quality and high fidelity for subsequent training of deep learning models. Combining such domain-specific preprocessing with pretrained convolutional models has been shown to improve classification performance on small medical-imaging datasets (37).

### 2.2 ROI labeling and dataset division

At the lesion level, 11 lung minimally invasive adenocarcinoma (MIA) and 16 lung adenocarcinoma *in situ* (AIS) lesions were randomly selected from the 225 lesions. Following the DICOM-to-PNG conversion, a total of 218 images were allocated as an independent test set. This test set was exclusively reserved for the visual evaluation of the final model's performance; it remained strictly independent throughout all subsequent processing stages and was entirely excluded from image augmentation and model training. Because each lesion was imaged on multiple contiguous thin-section (1 mm) CT slices, every slice on which the lesion appeared was exported as a separate PNG image; consequently, the 27 selected lesions yielded 218 test images (on average approximately eight images per lesion). As the pathological category is defined at the lesion level, all images originating from a given lesion share its label; because the number of qualifying slices differed between the two subtypes, the image-level class distribution of the test set (110 MIA and 108 AIS images, close to 1:1) does not mirror the lesion-level ratio (11 MIA : 16 AIS). The test-set precision and recall reported in Section 5.5 are accordingly computed per image and should be interpreted with the limited number of underlying lesions (*n* = 27) in mind.

The remaining lesions were similarly converted into PNG images and subsequently underwent data augmentation via horizontal flipping, ultimately yielding 3,000 images for the training and validation sets. These images were partitioned at an 8:2 ratio, with four folds serving as the training set and the remaining one fold as the validation set. To ensure the robustness of the model training, this partitioning and training procedure was repeated five times to generate five data subsets with distinct distributions, thereby facilitating a 5-fold cross-validation. In quantitative terms, the ~198 training lesions contributed on the order of 1,500 original axial slices (each lesion spanning several contiguous 1-mm sections), which horizontal flipping approximately doubled to the ~3,000 images used for cross-validation. Horizontal flipping is a deliberately conservative, label-preserving operation; its limited diversity, and the fact that the flips were generated before the folds were partitioned, are addressed in the Limitations.

Two experienced physicians used LabelImg to label the training and validation sets. The specific operation process is as follows: import the preprocessed and converted CT images into PNG format into the software; draw a rectangular frame for the nodule area according to the lesion boundary, and assign the corresponding category label to each target. After the annotation is completed, the software automatically outputs a label file with the same name as the image, recording the target category number and the normalized center coordinates, width and height information. The data format is consistent with the input specification of the YOLO model. The category label assigned to each bounding box was taken directly from the postoperative histopathological diagnosis, which served as the gold standard, rather than from the physicians' independent visual classification; the two physicians delineated the lesion boundaries and reconciled any discrepancies in box placement by consensus. Because the class labels were thus determined by pathology and the boxes were produced through this consensus procedure rather than by independent duplicate annotation, a quantitative inter-rater agreement coefficient (e.g., Cohen's kappa) was not computed; this point is acknowledged as a limitation in Section 6.

## 3 Construction of the YOLOv5s model

### 3.1 Network architecture and training parameters

**Figure 2:**
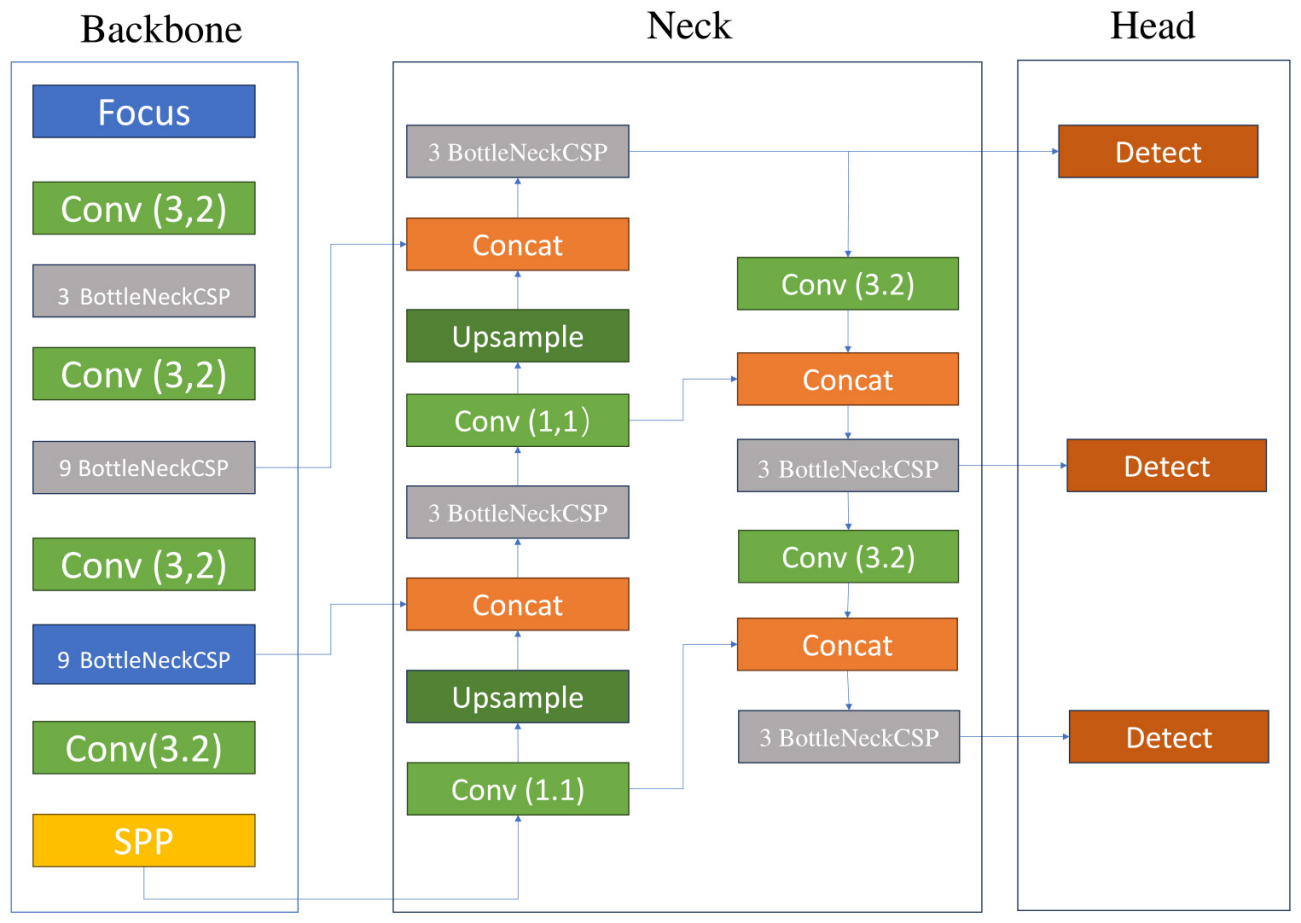
YOLOv5s structural diagram.

As shown in Figure 2, YOLOv5s is a lightweight single-stage object detection network in the YOLO series. Its overall structure consists of four modules: input terminal, backbone network, feature fusion network, and detection head.

The input end is responsible for unifying the size and grayscale of CT images, and applying regular data augmentation operations to ensure that the network receives input in a consistent format, thereby maintaining the robustness of the training process (38). After the image enters the backbone network, feature representations of different scales are generated through layer-by-layer convolutional coding. In this process, the network extracts semantic information while retaining key spatial details, laying the foundation for subsequent multi-scale feature fusion. The feature fusion network integrates shallow detailed information and deep semantic features based on backbone features. Through cross-layer information interaction, the network's ability to perceive targets of different sizes is enhanced, and the response to small targets and low-contrast lesions is also improved.

The fused multi-scale features are ultimately fed into the detection head to output the bounding box coordinates and class probabilities for each candidate region, thereby accomplishing end-to-end detection inference. This architecture maintains high detection accuracy while preserving inference speed, making it well-suited for the rapid automated identification of lung nodules and AIS/MIA lesions in clinical CT data. The overall network pipeline is highly efficient and straightforward to implement. It provides a stable and viable network baseline for the future integration of attention mechanisms or advanced feature fusion methods, and lays a solid technical foundation for the clinical application of the end-to-end automated identification of small lesions.

The principal training hyperparameters are summarized here, in keeping with the section title, and are reported together with the full hardware and software environment in Section 5.2: all models were trained for 100 epochs with the SGD optimizer, a batch size of 8, an initial learning rate of 0.01 (1 × 10^−2^), and an IoU matching threshold of 0.6.

### 3.2 Analysis of shortcomings of baseline model

YOLOv5s performs well in balancing lightweight and real-time inference, but it still exposes several structural limitations when directly applied to the detection of small targets and low-contrast lesions (39).

Regarding feature preservation, although the multi-scale fusion mechanism integrates features across different levels, shallow detailed information is prone to attenuation during successive downsampling operations. Consequently, the edge signals of extremely small nodules and the localized solid components within minimally invasive regions often fail to elicit effective responses in the deep layers of the network. Simultaneously, the network lacks the capacity for active, selective suppression of noise and non-lesion regions within the images. This deficiency renders it susceptible to false positives in complex backgrounds, or prone to missed detections of low-contrast lesions. These issues potentially compromise the classification accuracy between AIS and MIA. Collectively, these challenges highlight the necessity of introducing a targeted feature enhancement mechanism into the existing framework to compensate for the baseline model's inadequacies in fine-grained lesion recognition tasks.

To address these challenges, this study aims to incorporate an attention mechanism to amplify the network's responsiveness to minute solid structures and minimally invasive regions within the nodules. Concurrently, by coupling this with an improved multi-scale feature fusion strategy, shallow details can be effectively preserved and the utilization efficiency of deep semantic features enhanced, thereby improving small object detection capability and AIS/MIA classification accuracy. This trajectory of improvement not only delivers targeted optimization for the performance limitations of YOLOv5s, but also provides technical support for the practical application of lightweight detection networks in the automated identification of clinical lung nodules.

## 4 Feature fusion and attention mechanism improvement experiments

### 4.1 Innovation and improvement ideas

The task of detecting pulmonary nodules itself is quite difficult, especially for AIS/MIA lesions. The lesion area occupies a very small proportion in the image, and contains both low-contrast features and micro-infiltration structures inside, making the network prone to response bias during feature extraction, tending to capture large-area, high-contrast areas, while ignoring the subtle nodule features that are also critical in pathology. In response to this inherent bias, this study adopts a step-by-step improvement and innovation strategy. Through modular ablation design, the actual contribution of each improvement component to feature representation quality and overall detection performance is evaluated one by one.

In order to test the role of feature fusion in enhancing small target recognition capabilities, we first conducted a global replacement experiment on the original multi-scale fusion module of YOLOv5s: BiFPN or ASFF was applied to the entire network to observe the improved effect of the network on candidate area recognition and feature integration. Then, further local replacement experiments were conducted to improve only the low-level features corresponding to the P3 detection head to optimize the attenuation problem of small target features. While retaining high-level semantic information, this strategy makes the network more responsive to tiny nodules, verifying the key role of low-level features in tiny object detection.

Although multi-scale feature fusion broadens the network's perception range of targets of different sizes, it fails to fundamentally solve the problem of insufficient information selectivity in low-contrast areas. The micro-solid component and micro-infiltration boundary within the nodule have weak signals in CT images, and the contrast with the surrounding tissue is low. It is difficult for the network to generate sufficient feature activation by relying solely on multi-scale fusion. In response to this problem, this study introduces the coordinate attention mechanism (Coordinate Attention, CA). CA performs adaptive pooling along two independent directions, horizontal and vertical, respectively, encoding spatial position information into direction-aware attention weights, and acts on the original feature map with element-wise multiplication, thereby introducing clear spatial coordinate awareness beyond the channel dimension. The network is therefore able to form an accurate response to the position of the target in the row and column, rather than treating the entire feature map equally.

The directional encoding mechanism of CA concentrates attention resources on the spatial coordinate regions actually occupied by the lesions, yielding a targeted enhancement of the activation responses to the internal microstructural features of the nodules, while simultaneously suppressing redundant activations in non-lesion background regions. This characteristic is particularly critical for localizing the candidate regions of AIS and MIA lesions. As detection sensitivity improves, the false positive rate induced by background interference is correspondingly reduced, thereby providing higher-quality feature inputs for subsequent classification decisions.

On the basis of preliminary experiments, the local contrast-spatial attention module [Local Contrast-Spatial Attention (LCSA)] and its upgraded version [Local Contrast-Spatial Attention v2, (LCSAv2)] were innovatively designed based on CA to further improve the network's information selectivity in shallow details and deep semantic features, reduce noise interference, and provide a more robust feature response for micro-nodule detection.

Finally, LCSA/LCSAv2 was combined with BiFPN and ASFF to form a multi-module collaborative optimization solution. The network achieves the collaborative integration of shallow detail features, deep semantic information, and attention-weighted features while maintaining lightweight and real-time performance. This combined design not only improves the recall rate of micro-nodule detection, but also provides more accurate candidate areas and high-quality feature representations for AIS/MIA classification, forming a complete optimization path from the baseline model to multi-module improvement.

### 4.2 BiFPN feature fusion

**Figure 3:**
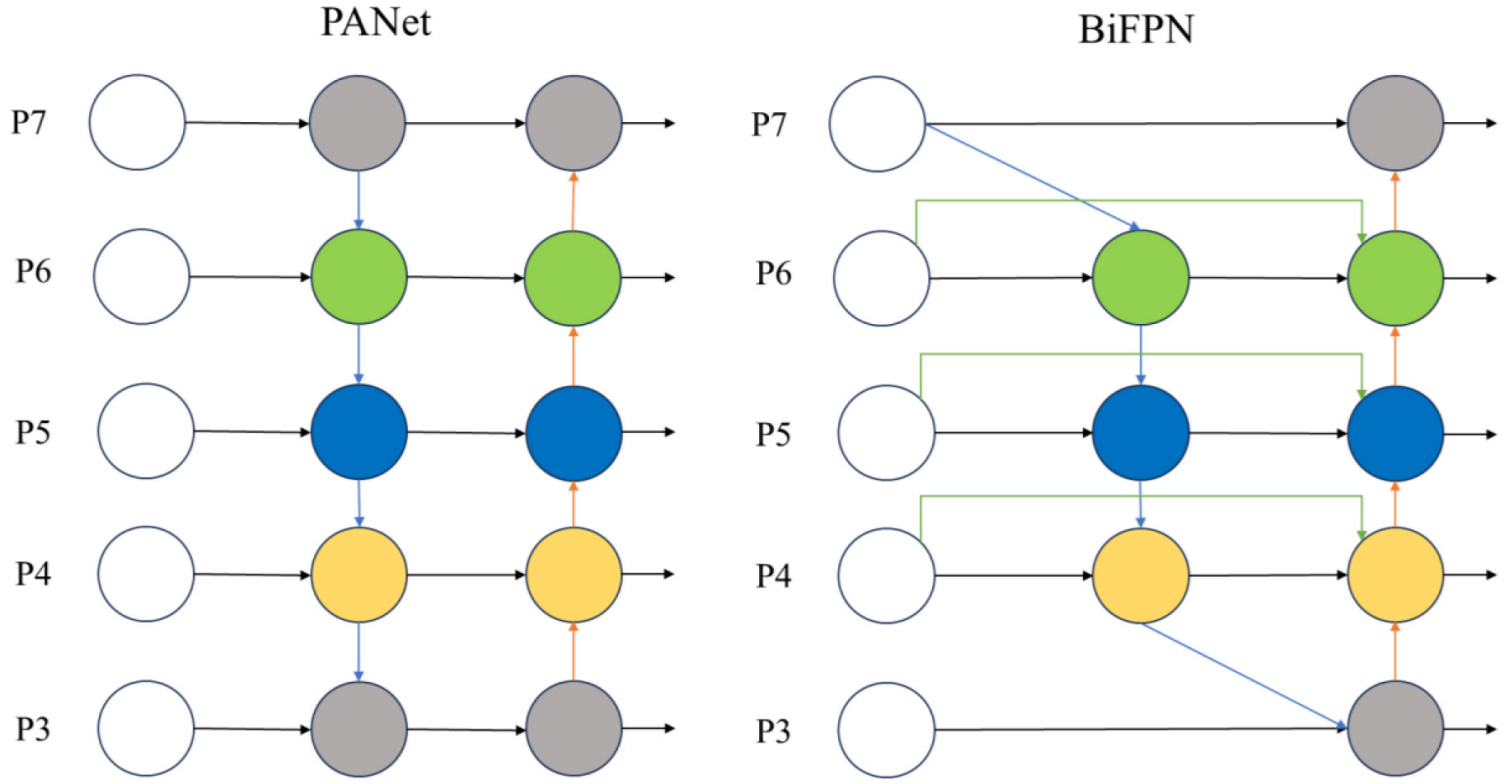
Comparison diagram of PANet and BiFPN structures.

The original YOLOv5 uses the PANet structure as the feature fusion module (shown on the left side of Figure 3). Its fusion path consists of two one-way paths, top-down and bottom-up, connected in series (24). In the top-down path, high-level semantic features are upsampled and fused with low-level features step by step to complete the downward transmission of semantic information; then, in the bottom-up path, low-level features are downsampled and fused upward again step by step to form a two-way feature transfer chain. However, the two paths of PANet are strictly one-way transfer step by step. Each feature layer only receives information input from adjacent layers, and there is a lack of direct connection channels between cross-layer features. In addition, the fusion weights of each layer are fixed and cannot be adaptively adjusted according to the feature requirements of targets of different scales.

In order to solve the problem of PANet's step-by-step transfer limitation, this study introduced the Bi-directional Feature Pyramid Network (BiFPN), and on this basis introduced a cross-layer direct connection and weighted fusion mechanism (shown on the right side of Figure 3). Compared with PANet, BiFPN adds additional cross-layer skip connections in addition to the standard bidirectional path, allowing direct information interaction between non-adjacent feature layers, such as cross-level connections between P3 and P5, P6 and P7, thus breaking the strict level-by-level transfer restrictions in PANet (28). In addition, BiFPN assigns learnable fusion weights to each input feature, allowing the model to dynamically determine the contribution ratio of different layer features based on the scale characteristics of the current target, rather than splicing or summing all feature layers equally.

By incorporating cross-scale skip connections and a weighted feature fusion mechanism, BiFPN facilitates a more efficient bi-directional information flow. Specifically, the newly added cross-layer connections provide a direct conduit for shallow detailed information to reach the deep feature maps. This effectively mitigates the information attenuation of small object features during successive downsampling operations, thereby preserving richer spatial structural cues for subsequent bounding box regression. Simultaneously, the top-down pathway enables the reverse injection of deep semantic information into the shallow layers, providing the global context necessary for distinguishing fine-grained categories such as AIS and MIA. More crucially, BiFPN assigns learnable fusion weights to each input feature, enabling the network to dynamically adjust the contribution proportions of features from different levels based on the target scale and imaging characteristics of the current nodule. Consequently, the internal solid components and minimally invasive structures of the nodules receive feature response weights commensurate with their discriminative value, thereby enhancing the model's capacity to differentiate between distinct pathological subtypes.

In the present study, the integration of the BiFPN module followed a progressive validation and optimization strategy. Initially, to evaluate the impact of multi-scale feature fusion on the overall network, a global replacement experiment was conducted. Designated as YOLOv5s-BiFPN, this configuration replaced the original fusion modules across all detection heads of YOLOv5s with BiFPN. Furthermore, a local replacement strategy was proposed, wherein only the P3 detection head—which is responsible for the low-level features of small objects—was replaced with BiFPN; this configuration was designated as YOLOv5s-BiFPN@P3. This strategy was designed to leverage the P3 detection head's sensitivity to minute objects, validating that the isolated optimization of small object features aligns more closely with the specific task requirements of this study than a global replacement approach.

### 4.3 ASFF multiscale adaptive fusion

**Figure 4:**
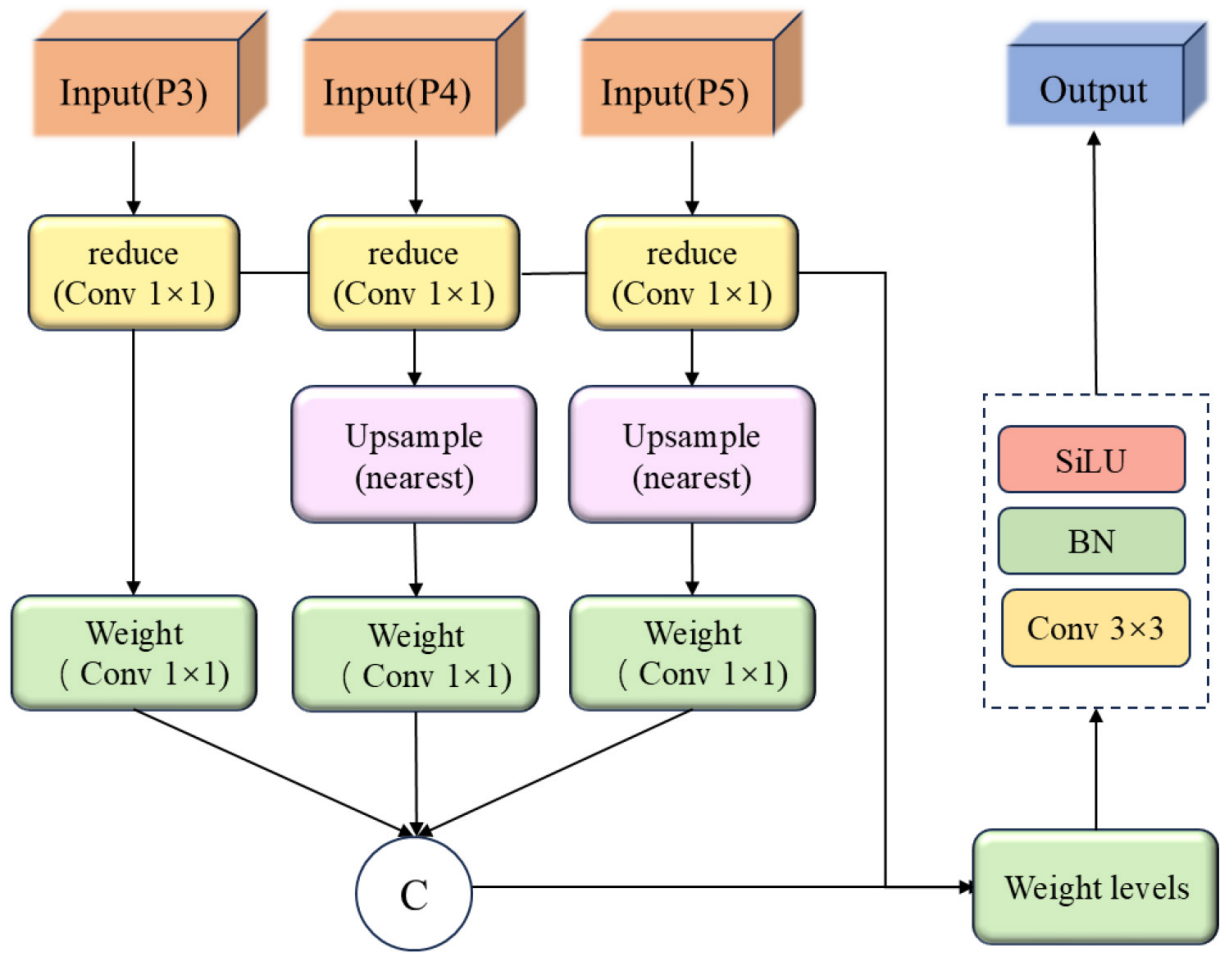
Schematic diagram of ASFF structure.

Adaptive Spatial Feature Fusion (ASFF) is a multi-scale feature fusion architecture whose core design lies in the spatially adaptive integration of features across different scales. It facilitates the dynamic allocation of weights from feature maps of varying resolutions to each spatial pixel location, as illustrated in Figure 4 (the schematic diagram of the ASFF module structure).

In ASFF, feature maps from different scales are aligned to a unified resolution through upsampling or downsampling, and then fusion weights are generated based on spatial positions, and the features of each scale are weighted and summarized, thereby retaining the most informative features between different scales while suppressing redundant or irrelevant information (40). Taking into account the requirements for inference speed in clinical deployment, the lightweight nature of the ASFF module can be seamlessly embedded into the YOLOv5s framework, which improves multi-scale feature fusion capabilities while causing almost no additional computing burden.

The ASFF module adaptively selects the most contributing scale features for each spatial position, so that the shallow fine-grained texture and edge information and the deep semantic information are optimally integrated spatially. This mechanism enhances the network's ability to perceive micro-nodules and their internal micro-infiltration structures, ensuring that small target features are not diluted during deep transmission, thereby improving the performance of AIS/MIA lesion location and classification.

In this study, the placement of ASFF also followed a stepwise verification and optimization strategy. First, to evaluate the impact of multi-scale spatial adaptive fusion on overall network performance, we conducted a global replacement experiment: replacing the original fusion modules of all YOLOv5s detection heads with ASFF, and named the experiment YOLOv5s-ASFF. After that, in order to further highlight the perception effect of tiny nodules, we proposed a local replacement strategy: only replace the P3 detection head responsible for small target features with ASFF, and named the experiment YOLOv5s-ASFF@P3. This strategy makes full use of the sensitivity of the P3 detection head to small targets and significantly improves the detection performance of small nodules without increasing the overall calculation amount. At the same time, it is verified that optimizing small target features individually is more in line with the requirements of this research than global replacement.

In addition, the introduction of the ASFF feature fusion module also aims to eliminate the possibility that other feature fusion is effective in improving model performance, and provides a reasonable experimental basis for the subsequent embedding of the attention mechanism in the P3 detection head.

### 4.4 CA attention mechanism

**Figure 5:**
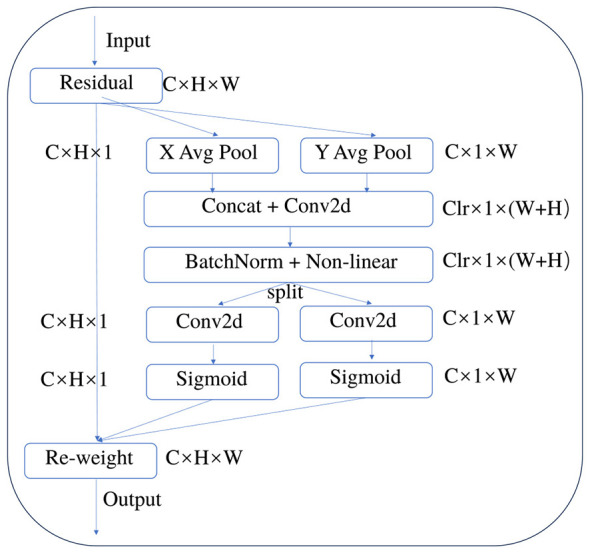
Schematic diagram of CA attention mechanism structure.

Coordinate Attention (CA) was proposed by Hou et al. (41), is a lightweight attention mechanism that simultaneously encodes channel information and spatial direction information. It can encode channel information and spatial direction information at the same time, as shown in Figure 5 (which presents a schematic diagram of the CA module structure). CA performs adaptive average pooling along the horizontal and vertical directions respectively, decomposes the feature map into two direction encoding vectors, and retains spatial position information while compressing channel redundancy. The two-way vectors are jointly encoded through splicing, convolution, batch normalization and non-linear activation. They are then independently convolutionally decoded and bidirectional attention weights are generated through Sigmoid normalization. The original feature map is spatially calibrated by element-by-element multiplication.

In order to highlight the design advantages of CA, compared with the design of SENet, which only focuses on channel dimensions and spatial attention only generates global saliency maps, CA takes into account the dual perception capabilities of channels and space with a small number of parameters and low inference overhead, and is especially suitable for the spatial localization task of small lesions. AIS and MIA lesions are small in size, have fuzzy boundaries, and contain both micro-solid components and mild infiltrative structures inside, which places high requirements on the accuracy of feature extraction. CA accurately locates the network attention to the coordinate area where the lesion is located through directional spatial coding, and at the same time dynamically enhances the channel features related to the lesion, thereby suppressing the interference of background noise, thereby improving the perception of small nodules and micro-infiltration areas.

Based on the aforementioned feature fusion experiments, in order to optimize the detection performance of micro-nodules, this study embeds the CA module separately into the P3 detection head to make full use of the detection head's high sensing ability for micro-nodules. However, the design of CA mainly focuses on channel dimensional characteristics, and the fine microstructural response to spatial position is still limited. To make up for this limitation, this study proposes the Local Contrast-Spatial Attention (LCSA) module. Through lightweight collaborative design that combines spatial and channel attention, LCSA further enhances the expression of internal microstructural features of nodules while remaining lightweight, effectively improving small target recognition and subtype classification performance.

### 4.5 LCSA attention mechanism design

**Figure 6:**
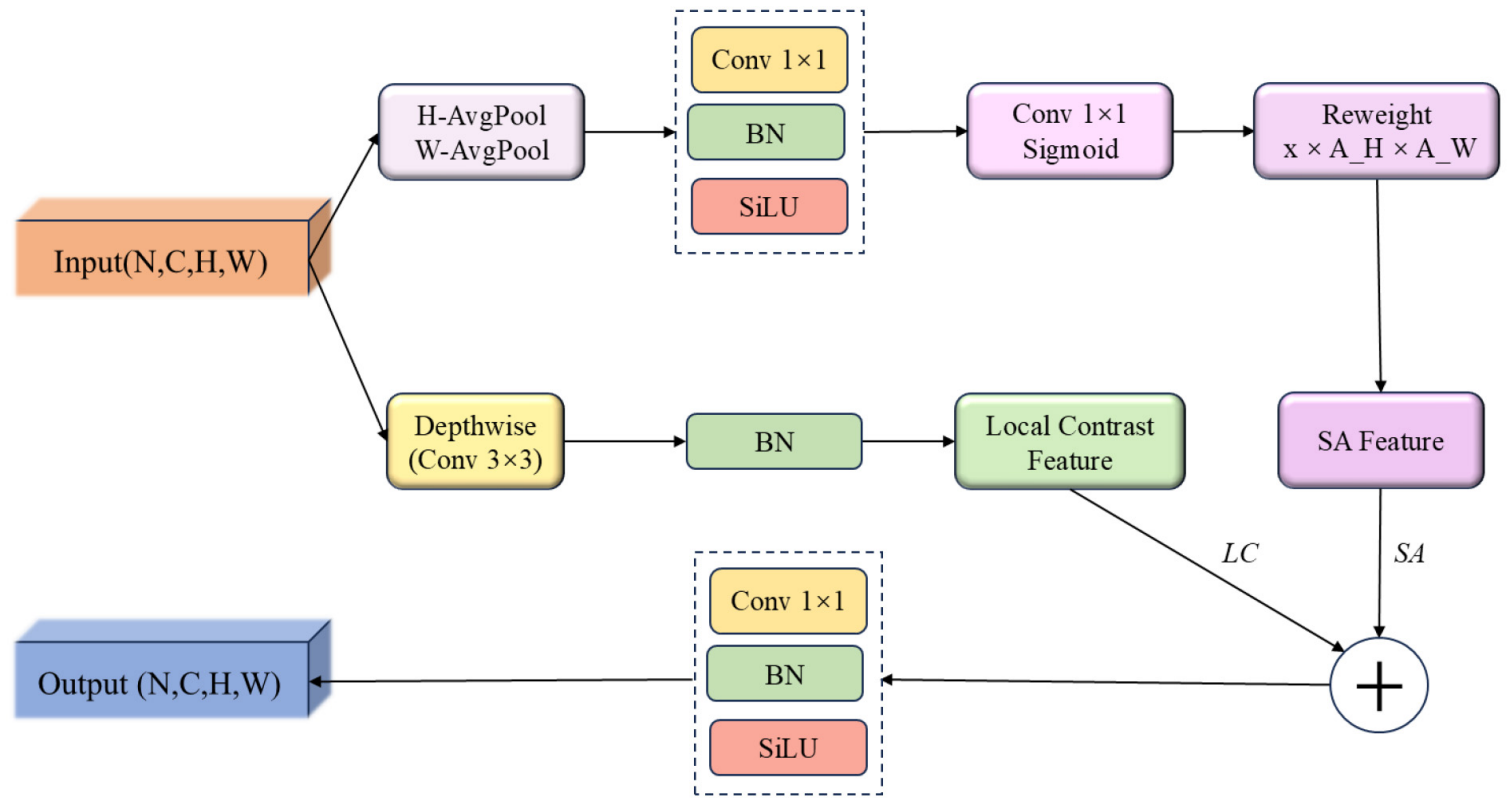
LCSA structural diagram.

Figure 6 is a schematic diagram of the LCSA module structure. The LCSA module adopts a dual-branch parallel structure, which is composed of a local contrast branch and a spatial attention branch. The two-channel features are additively fused to complete information reintegration, providing a unified feature representation for subsequent channel reconstruction.

The local contrast branch uses depth-separable convolution to perform convolution operations on each channel independently, and extracts local neighborhood structure information without basically increasing the number of parameters. Afterwards, the batch normalization layer stabilizes the feature distribution to ensure numerical consistency during the training process. In parallel with this, the spatial attention branch receives the same original input, uses the coordinate attention mechanism to encode spatial position information along the horizontal and vertical directions respectively, generates a direction-aware attention weight map, and explicitly models the spatial importance of the potential target area. The outputs of the two branches are additively fused and integrated into a unified representation, and the structural details after local enhancement and the spatial dimension weight modulation results are retained at the same time. The fused features are then subjected to 1 × 1 convolution, normalization, and non-linear activation to complete channel reconstruction, and the final output is an enhanced feature with both local contrast sensitivity and spatial position awareness. This design allows the nodule edges on the feature map, which were originally submerged by background noise, to generate a stronger gradient response after passing through the LCSA module. The spatial attention ensures that these enhanced responses can accurately fall on the nodule center area and finally enter the detection head. The core of the lesion is preserved while the edges are also identified.

The core function of LCSA is to implement structure-sensitive enhancement of small low-contrast targets. Pulmonary nodules are mostly characterized by small grayscale changes and gentle boundary transitions in CT images. The target signal is easily overwhelmed by the smooth background when propagating in deep networks. To address this problem, the local contrast branch uses learnable convolution operations to model neighborhood pixel differences, strengthen local gradient changes, and improve the high-frequency boundary response in the lesion area. At the same time, the low-frequency redundant components of the smooth background are suppressed, ensuring that the expression intensity of weak signals cannot be diluted in multi-layer propagation. On this basis, the spatial attention branch assigns higher feature weights to enhanced structural areas globally, while suppressing activation of irrelevant backgrounds, forming a two-stage enhancement logic of first enhancing the signal, then optimizing the distribution.

Compared with representative attention mechanisms commonly used in current research, including SE, CBAM, BAM, and ECA-Net (31, 42–44), existing methods tend to emphasize feature weighting rather than active feature enhancement when processing low-contrast small nodules such as AIS and MIA. The architecture of LCSA is specifically devised to bridge this gap. The paramount objective of its local contrast branch is to explicitly delineate and sharpen the blurred boundaries of nodules prior to the weighting process. Consequently, this design is inherently task-driven for scenarios involving ground-glass nodules (GGNs) like AIS and MIA, rather than constituting a mere straightforward transplantation of generic attention mechanisms.

To position the proposed module precisely, LCSA is contrasted here with the representative Convolutional Block Attention Module (CBAM). CBAM operates primarily on a “feature weighting” paradigm; it derives its spatial attention map from channel-pooled descriptors (global average and max pooling) passed through a standard convolution. Consequently, the resulting attention merely re-weights an already-smoothed feature map, offering no explicit enhancement of weak boundary gradients. For sub-centimeter ground-glass nodules (GGNs), whose discriminative signals are inherently faint and low-contrast, this pure-weighting mechanism fails to restore the blurred edges separating the lesion from the surrounding parenchyma.

The defining difference of LCSA is that it couples feature enhancement with feature weighting, rather than performing weighting alone. Its local-contrast branch applies a learnable depth-wise convolution that explicitly models centre-vs.-neighborhood gray-level differences, actively sharpening the blurred nodule boundaries before attention mapping. A parallel directional attention branch then concentrates these enhanced responses on the true lesion coordinates. In short, where CBAM answers “how to weight existing features,” LCSA operates on a “first enhance the weak signal, then redistribute it” logic. This mechanism is theoretically better matched to pulmonary GGN detection, as the primary challenge in identifying early-stage lung adenocarcinoma is the dilution of low-gradient lesion boundaries during successive down-sampling—a vulnerability that demands active feature enhancement rather than generic re-weighting.

### 4.6 LCSAv2 improved design

**Figure 7:**
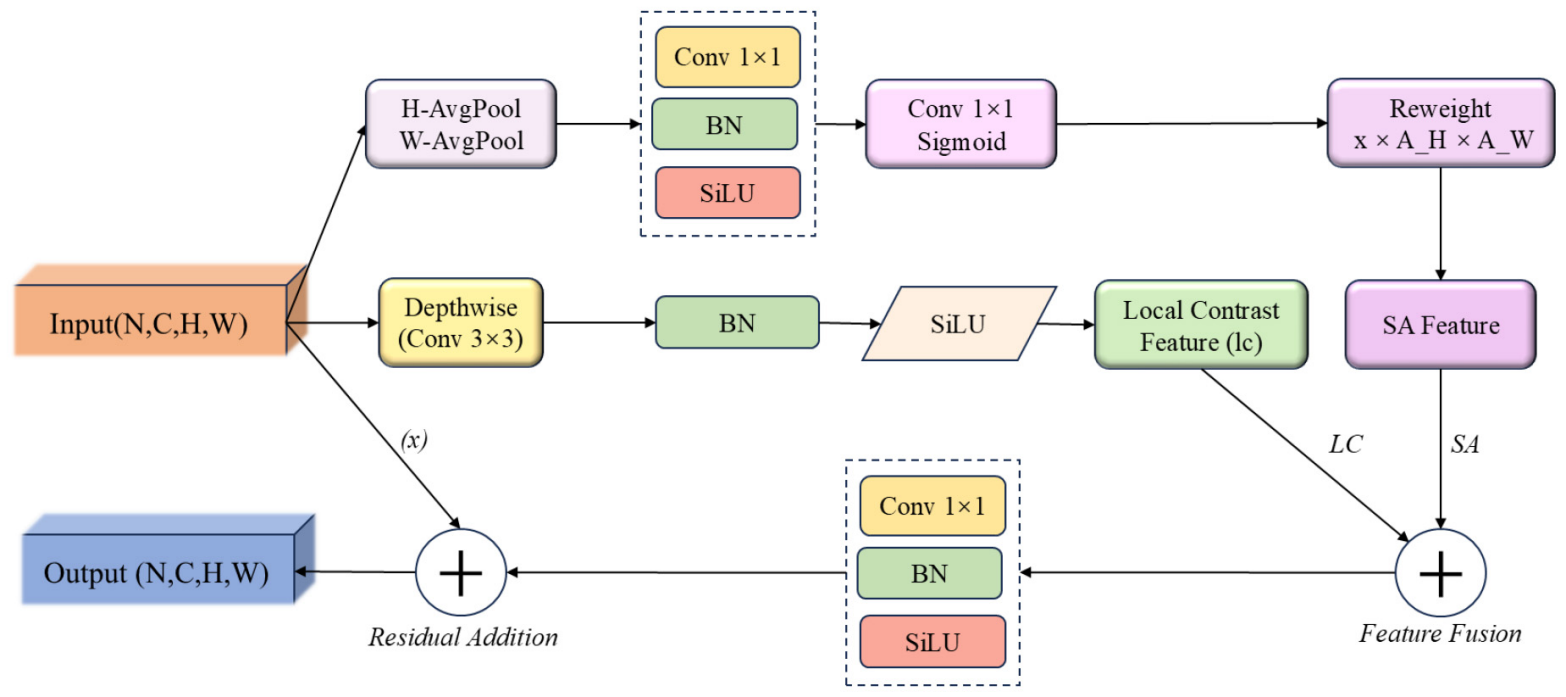
LCSAv2 structural diagram.

Figure 7 is a schematic diagram of the LCSAv2 module structure. LCSAv2 continues the dual-branch parallel design in the overall architecture. The basic composition of the local contrast branch and spatial attention branch remains consistent with the original LCSA. The core changes focus on the two structural details and the final residual connection mechanism.

The local contrast branch still performs channel-by-channel convolution with depth-separable convolution to extract local neighborhood structure information. Different from the original version, v2 inserts the SiLU non-linear activation layer after the convolution operation, so that the local enhanced features can complete independent non-linear mapping before entering the fusion stage. This adjustment improves the separability of local branch feature distribution, allowing the two branches to form a more complete feature representation before fusion. The spatial attention branch retains the coordinate attention structure, encodes spatial position information along the horizontal and vertical directions, generates direction-aware attention weights, and implements adaptive emphasis on key areas. After the two outputs are additively fused, channel reintegration is completed through 1 × 1 convolution, batch normalization and non-linear activation.

The most essential structural difference between LCSAv2 and LCSA is the introduction of residual connections. The original LCSA directly outputs the fused feature map, while v2 adds the original input x and the fusion result element by element to form a standard residual expression. This change allows the module to only learn the correction amount superimposed on the original features, ensuring the integrity of the original semantic information and reducing the risk of expression distortion caused by excessive enhancement or feature offset.

Therefore, compared with LCSA, the innovation of LCSA v2 does not lie in the change of the branch structure, but in optimizing the information flow path through the residual mechanism, so that local contrast enhancement and spatial attention modulation have stronger stability and trainability while maintaining expression ability, thereby improving the performance of micro-nodule recognition and AIS/MIA subtype classification.

## 5 Ablation experiment and high IoU performance analysis

### 5.1 Single module and combined module ablation experiments

**Figure 8:**
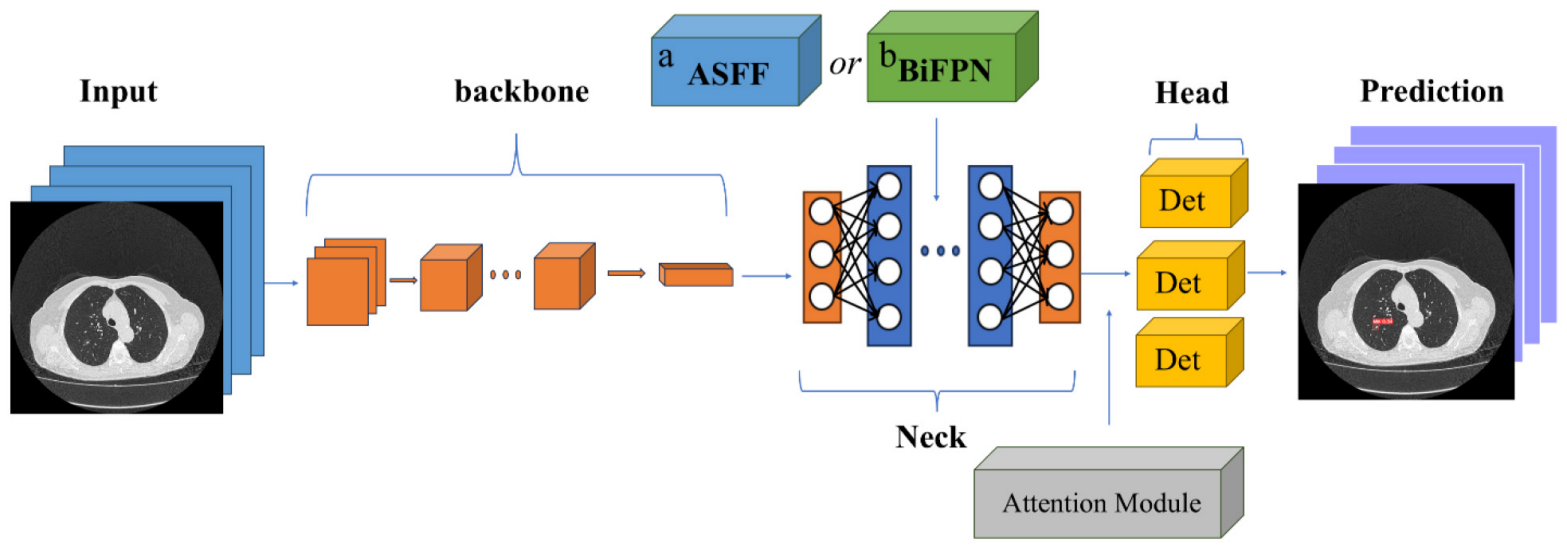
Insertion positions of the modules.

Figure 8 presents the overall design idea of module embedding and structural improvement in this study. ASFF and BiFPN are used as feature fusion modules to replace the fusion structure of the Neck part of the original YOLOv5s network. This design retains the hierarchical structure of the original detection framework, and only implements targeted enhancements to the multi-scale fusion path, which improves the model's detection performance for small targets and complex background conditions.

LCSA and LCSAv2 are embedded in the position after the C3 module of the P3 detection head and before the input of the detection layer to further enhance the features of small targets. The P3 detection head has the highest spatial resolution, and its ability to perceive the characteristics of tiny nodules is stronger than other levels. The lower-resolution P4 and P5 detection heads, whose feature maps have undergone additional downsampling and therefore carry coarser spatial detail for sub-centimeter nodules, were consequently not chosen as the insertion point. This decision rests on the spatial-resolution and information-loss rationale stated above rather than on an empirical comparison of insertion positions; a positional ablation (e.g., LCSA at P4 or P5) was not performed in this study and is noted as a limitation in Section 6. The attention mechanism deployed here can exert maximum effectiveness on nodes with minimal information loss. The features enhanced by attention then enter the subsequent multi-scale fusion module. Single-scale fine enhancement and cross-scale adaptive fusion form a synergy and work together to improve the final detection performance. In order to explore the impact of the two improved modules on model performance, an ablation experiment was designed. The specific experimental plan and combination configuration of each module are shown in Table 1 below.

**Table 1:** Baseline model selection and evaluation of improved network architectures.

Model structure	Experimental design type	Research objective
Baseline (YOLOv5s)	Controlled experiment	Establish a baseline of original model performance to provide a reference standard for all structural improvements
BiFPN (full replacement of Concat)	Fusion structure replacement	Verify the impact of bidirectional feature pyramid structure on multi-scale information flow reconstruction
ASFF (full replacement of Concat)	Fusion structure replacement	Evaluate the improvement effect of adaptive spatial feature fusion mechanism on multi-scale weighted allocation capabilities
BiFPN @ P3	local fusion enhancement	Analyze the impact of introducing a bidirectional fusion structure only in the shallow feature layer on small object detection
ASFF @ P3	local fusion enhancement	Analyze the impact of introducing an adaptive multi-scale weighting structure only in the shallow feature layer on small object detection
CA @ P3	single attention ablation	Verify the enhanced ability of traditional coordinate attention mechanism in shallow semantic features
Local Contrast @ P3	local enhanced ablation	Analyze the contribution of individual local contrast enhancement to the expression ability of weak contrast targets
LCSA @ P3	Attention fusion verification	Verify the synergistic enhancement effect of the joint mechanism of local contrast and spatial attention
LCSA × BiFPN	Module collaboration analysis	Explore the complementary mechanism between single-scale enhancement and bidirectional multi-scale fusion structures
LCSA × ASFF	Module collaboration analysis	Evaluate the joint effect of local enhancement mechanism and adaptive multi-scale weighted fusion
LCSA v2	Structural improvement verification	Verify the impact of residual enhancement design on feature stability and information retention capabilities
v2 × BiFPN	Residual + fusion synergy	Analyze the stability and gain characteristics of residual enhanced attention in the bidirectional fusion structure
v2 × ASFF	Residual + fusion synergy	Evaluating the collaborative optimization capabilities of residual enhanced attention and adaptive weighted fusion mechanisms

### 5.2 Experimental parameters

This experiment was conducted on a computer system equipped with the following hardware: 13th Gen Intel(R) Core(TM) i7-13620H 2.40 GHz central processing unit (CPU), NVIDIA GeForce RTX 4060 Laptop graphics processing unit (GPU), and 16.0 GB random access memory (RAM). The software environment is Python 3.8.7, PyTorch 2.0.0, CUDA 11.8, used to build the YOLOv5s model.

As a relatively mature lightweight detection architecture, YOLOv5s has a simple structure and clear optimization space, which provides a clear foundation for modular improvements. However, considering that YOLOv5s was released earlier, this study introduced YOLOv11s (45) under matched experimental conditions as a recent lightweight baseline comparison. In the training phase, for YOLOv11s, YOLOv5s and their derived 11 improved models, the SGD optimizer was used. The batch size was set to 8, the initial learning rate was 1 × 10^−2^, the number of training iterations was 100, and the IoU threshold was set to 0.6 to ensure the accuracy of candidate frame regression and target matching.

### 5.3 Experimental evaluation indicators

In the YOLOv5 model, precision, recall and mean average precision (mAP) are used to measure model performance (46). In this study, the model task was to distinguish adenocarcinoma *in situ* (AIS) from minimally invasive adenocarcinoma (MIA) in lung nodules. We set MIA as a positive class so that clinical scenarios can specifically explain the meaning of the evaluation indicators.

Precision indicates the proportion of nodules that are actually MIA among all the nodules predicted as MIA by the model. Specifically, when the model determines that a nodule is minimally invasive adenocarcinoma, what is the probability that this determination is correct. If the accuracy is higher, it means that the model misjudges AIS as MIA less frequently, that is, the number of false positives (False Positive) is low. At the clinical decision-making level, this means that the model is less likely to misclassify cases that should be adenocarcinoma *in situ* as minimally invasive adenocarcinoma.

The recall rate represents the proportion of all nodules that are actually MIA that are successfully identified by the model. That is, how many cases the model can detect in cases where microinvasive components actually exist. If the recall rate is high, it means that the model misses the diagnosis of MIA less frequently, that is, the number of false negatives (False Negative) is low. From a clinical point of view, this indicator is particularly important, because if a true MIA is misjudged as AIS, it may lead to insufficient treatment intensity or inappropriate follow-up strategies. Therefore, recall emphasizes the model's “ability to detect real high-risk cases.”

In short, in the identification task of AIS and MIA, precision reflects the reliability of the model's conclusion that “it is judged as MIA,” while recall reflects the model's ability to avoid missing the diagnosis of MIA. The former is related to whether there is excessive intervention, and the latter is related to whether risk cases are missed. The two jointly determine the safety and practical value of the model in clinical application. The specific calculation formulas are as follows (Equations 1 and 2).


$$\begin{array}{l} \text{Precision }=\frac{TP}{TP+FP} \end{array}$$ (1)

$$\begin{array}{l} \text{Recall }=\frac{TP}{TP+FN} \end{array}$$ (2)

The mean Average Precision at an IoU threshold of 0.5 (mAP@50) serves as the standard evaluation metric in object detection tasks. Under this metric, a True Positive is defined when the Intersection over Union (IoU) between the predicted bounding box and the ground truth is no less than 0.5, based on which the area under the Precision-Recall (PR) curve is calculated and averaged across all categories. In this study, the evaluation matching threshold was consistently fixed at 0.5, and mAP@50 was adopted as the unified detection performance indicator throughout the manuscript. Crucially, the non-maximum suppression (NMS) IoU threshold (iou-thres) was sequentially adjusted within a range of 0.6 to 0.9 to investigate the model's performance stability under varying degrees of post-processing stringency. This parameter is conceptually distinct from the evaluation matching threshold and must not be conflated. Furthermore, the anchor matching threshold during the training phase was set to 0.6, while the NMS IoU threshold was tuned independently during the inference phase, and the evaluation matching threshold remained strictly at 0.5; the definitions and functions of these three thresholds are entirely independent. The mAP and AP formulas are given in Equations 3, 4, where *p* is precision, *r* is recall and *N* is the number of classes.


$$\begin{array}{l} \int_{0}^{1}p\left(r\right)dr \end{array}$$ (3)

$$\begin{array}{l} mAP=\frac{\sum AP}{N} \end{array}$$ (4)

On the basis of the above evaluation indicators, in order to compare the required computing resources between different models, the evaluation indicator GFLOPs is introduced, which is a common indicator to measure the calculation amount of deep learning models. The higher the GFLOPs value of a model, it means that it requires more computing resources and may require more powerful hardware to support real-time or near-real-time inference (47).

### 5.4 Performance comparison under varying NMS IoU thresholds ranging from 0.6 to 0.9

The mean mAP@50 values for all compared models at an NMS IoU threshold of 0.6 are listed in Table 2. Overall, the performance gap between the improved models is relatively limited, but the design emphasis of different structures has initially shown a trend of differentiation. In the baseline model comparison, YOLOv5s performed comparably to YOLOv11s in precision, recall and mAP, while its GFLOPs (16.0) are about 25% lower than YOLOv11s (21.3). It maintains performance while having lower computing overhead, which is in line with the design goal of lightweight improvement in this study. After systematic improvements to the module, the model performance further exceeded the baseline level of YOLOv11s, indicating that the model still has the potential to compete with the new architecture after targeted optimization. Therefore, in the subsequent analysis, YOLOv11s will no longer be included, and instead we will focus on the systematic improvement of YOLOv5s performance by each improvement module. This baseline comparison rests on a single training run per architecture under matched settings; run-to-run variance was not characterized, so it is used only to justify adopting the mature, widely extended and lightweight YOLOv5s as the improvement base, and not as a general claim of superiority over YOLOv11s. Several newer detectors (YOLOv8–v12) were not evaluated and are left to future work.

**Table 2:** Comparison of mean mAP@50 across different models at an NMS IoU threshold of 0.6.

Model name	Precision	Recall	mAP@50	GFLOPs
YOLOv5s	0.849	0.932	0.947	16.0
YOLOv11s	0.838	0.884	0.93	21.3
YOLOv5s- BiFPN(All)	0.839	0.944	0.95	16.0
YOLOv5s- ASFF(All)	0.839	0.935	0.945	21.1
YOLOv5s-BiFPN	0.834	0.950	0.950	16.2
YOLOv5s-ASFF	0.839	0.941	0.948	18.3
YOLOv5s- Local Contrast	0.846	0.934	0.946	16.0
YOLOv5s- CA	0.848	0.939	0.947	16.0
YOLOv5s-LCSA	0.834	0.946	0.945	16.2
YOLOv5s-LCSA × BiFPN	0.841	0.942	0.947	16.4
YOLOv5s-LCSA × ASFF	0.836	0.941	0.946	18.5
YOLOv5s-LCSAv2	0.84	0.942	0.950	16.4
YOLOv5s-LCSAv2 × BiFPN	0.837	0.944	0.947	16.4
YOLOv5s-LCSAv2 × ASFF	0.838	0.948	0.948	18.5

In response to the recommendation to compare against more recent lightweight detectors, we emphasize that a current-generation architecture, YOLOv11s (45), was trained from scratch under identical data, hyper-parameters and evaluation settings and is reported alongside the baseline in Table 2: YOLOv5s matched its precision, recall and mAP@50 while requiring about 25% fewer GFLOPs (16.0 vs. 21.3), which is the basis for retaining the mature and widely extended YOLOv5s as the improvement backbone. After targeted optimization, the LCSA × ASFF model further surpassed the YOLOv11s baseline on the detection-oriented metrics, indicating that task-specific enhancement of a lightweight backbone can be more effective for AIS/MIA than simply adopting a newer but heavier architecture. The model is additionally benchmarked against published AIS/MIA classifiers (dual-head ResNet and EfficientNet variants) in Table 3.

**Table 3:** Model performance comparison summary table.

Model name	Precision	Recall
EfficientNet-b0 2D	0.6263	0.6371
Dual-head ResNet_3D	0.6066	0.6662
Dual-head ResNet_3D_3F	0.6685	0.6511
Dual-head ResNet_3D_19F	0.6405	0.6362
YOLOv5s-LCSA	0.9460	0.9450
YOLOv5s-LCSA × BiFPN	0.9420	0.9470
YOLOv5s-LCSA × ASFF	0.9410	0.9460
YOLOv5s-LCSAv2	0.9420	0.9500
YOLOv5s-LCSAv2 × BiFPN	0.9440	0.9470
YOLOv5s-LCSAv2 × ASFF	0.9480	0.9480

From the perspective of local performance, the performance of YOLOv5s and its improved models and the BiFPN local replacement structure achieved higher recall and mAP under the premise that the computational complexity is basically the same, and the object detection ability is relatively outstanding; the distribution of the three indicators of precision, recall and mAP of LCSAv2 is relatively balanced, the computational overhead is also maintained at a reasonable level, and the stability of feature representation is reflected. Although the ASFF-related structure shows a certain improvement in some indicators, the amount of calculation increases accordingly, and the trade-off between performance gains and resource consumption deserves attention. A direct comparison of the global and P3-local configurations clarifies why the global variants are listed in Tables 1, 2. At IoU = 0.6, restricting the fusion replacement to the high-resolution P3 head performed comparably to or better than the corresponding global replacement on the detection-oriented metrics: YOLOv5s-BiFPN (P3) matched the mAP@50 of YOLOv5s-BiFPN(All) (0.950 vs. 0.950) with slightly higher recall (0.950 vs. 0.944), and YOLOv5s-ASFF (P3) exceeded YOLOv5s-ASFF(All) in both recall (0.941 vs. 0.935) and mAP@50 (0.948 vs. 0.945) while using fewer GFLOPs (18.3 vs. 21.1). The global variants are therefore retained as control conditions, demonstrating that the improvement originates from targeted enhancement of small-object features at the P3 head rather than from wholesale replacement of the fusion path; for this task, global replacement offers no performance advantage and, in the case of ASFF, is the more computationally costly option.

In order to further verify the performance of the improved model, as shown in Table 3, this study compared it with research results in related fields. There are researchers (48). It is proposed to evaluate the infiltration of pure ground-glass nodules based on dual-head ResNet technology, emphasizing the importance of multi-branch feature extraction in capturing the subtle pathological characteristics of nodules.

Although different studies have differences in dataset sources and sample distribution, from an algorithmic logic point of view, the LCSA module and BiFPN structure introduced in this study are consistent with the previous studies in terms of strengthening the weight of key spatial information and fusion of multi-scale features (See the appendix for the specific pseudocode). It must be stressed that these external figures were obtained on different datasets, class definitions and evaluation protocols, and that the present values in Table 3 are five-fold cross-validation results at IoU = 0.6 that are themselves affected by the augmentation leakage noted in Section 5.5; the comparison is therefore illustrative of design trends only and is not a valid head-to-head accuracy benchmark.

The comparison shows that, on its own dataset, this model attains higher nominal precision and recall for AIS/MIA discrimination while maintaining low computational complexity (GFLOPs); because the studies use different datasets and protocols, this is not a like-for-like accuracy comparison. This shows that the optimization strategy combining local contrast attention and weighted feature fusion can effectively improve the model's ability to distinguish small lung lesions and has strong clinical application potential.

An NMS IoU threshold of 0.6 implies that candidate boxes with an overlap exceeding 0.6 are merged for deduplication, representing a relatively stringent suppression of redundant boxes during post-processing. Consequently, the evaluation primary focuses on establishing the baseline detection performance of the model under standard post-processing conditions. However, the performance under loose thresholds is difficult to fully reflect the model's true capabilities at the fine positioning level. To this end, it is necessary to extend the evaluation range to the continuous interval of IoU = 0.7 to 0.9, examine the frame regression quality and discriminant stability of each structure under more stringent positioning constraints, and reveal the performance differences of different improvement strategies.

**Figure 9:**
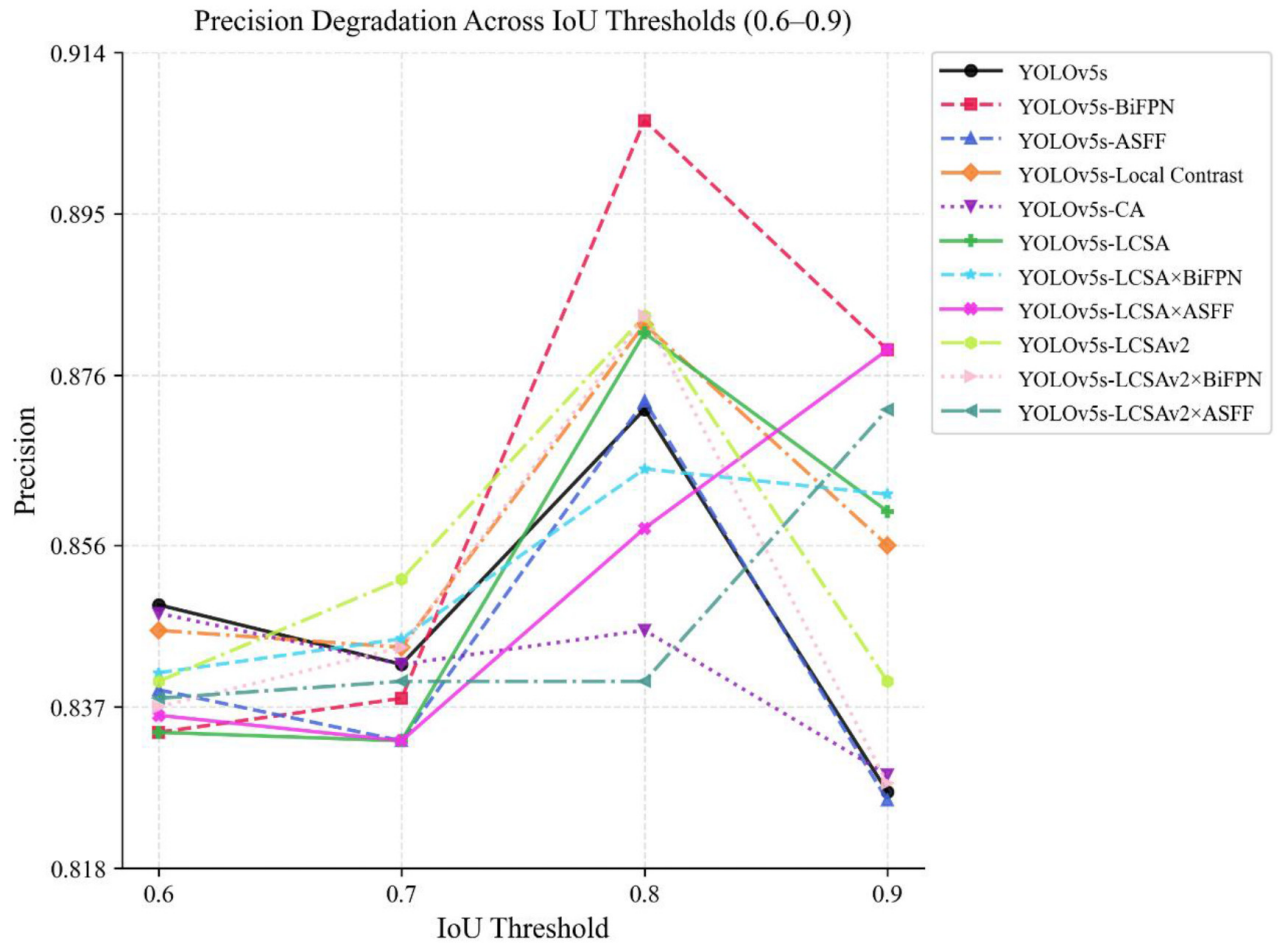
Accuracy attenuation curve under IoU = 0.6–0.9.

**Figure 10:**
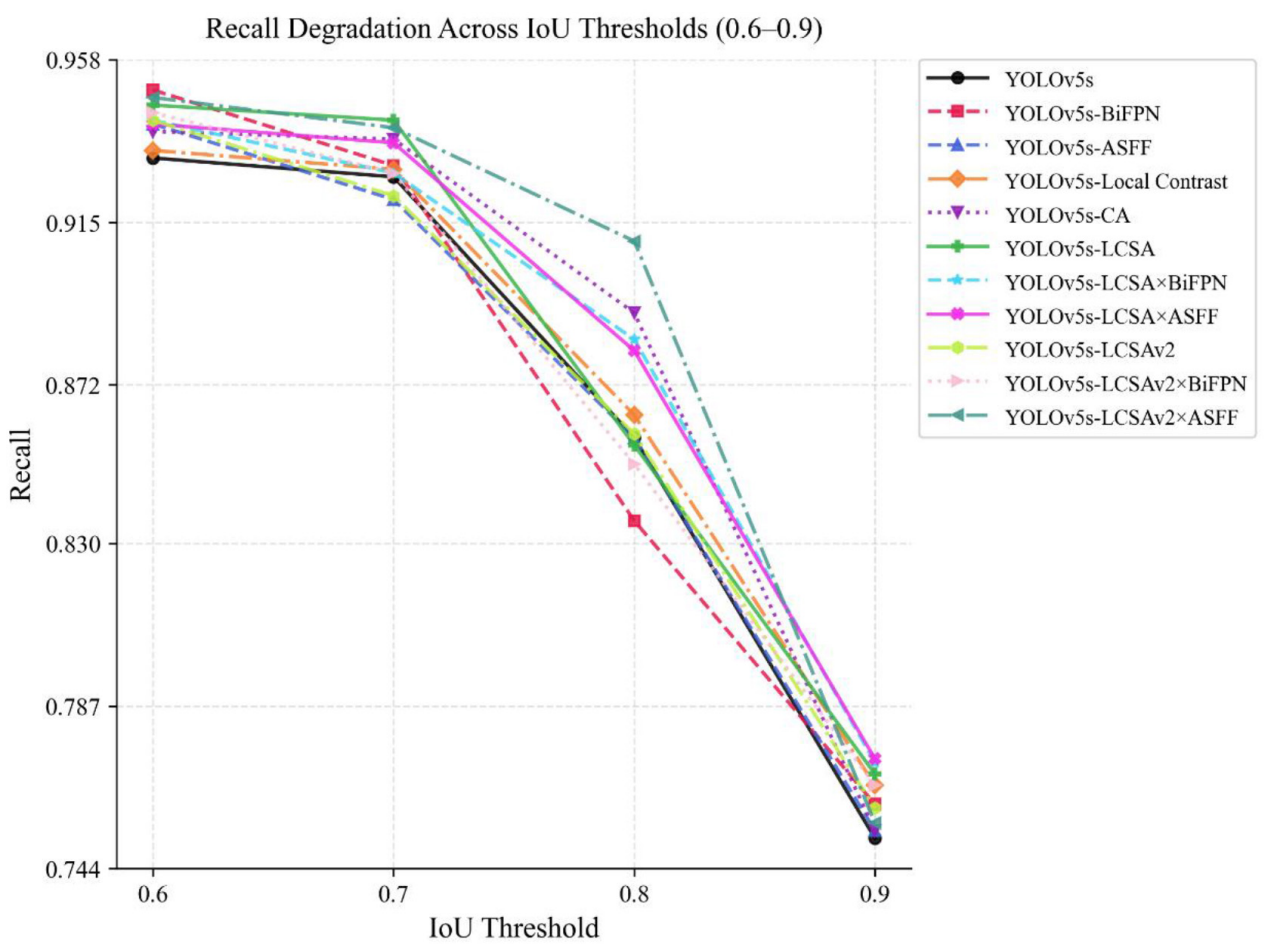
Recall decay curve under IoU = 0.6–0.9.

**Figure 11:**
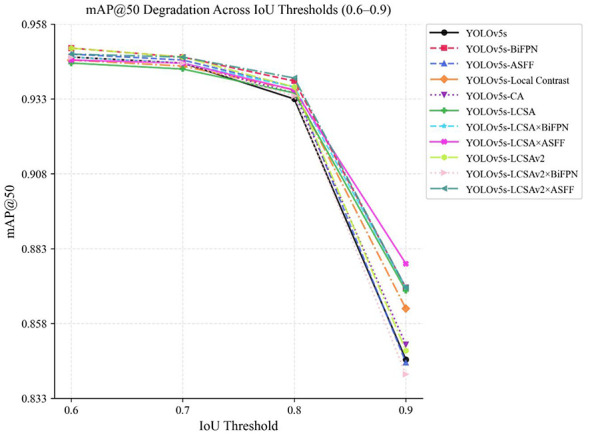
mAP attenuation curve under IoU = 0.6–0.9.

Tables 4–6 list the evaluation index values of each model under IoU = 0.7 to 0.9. Because GFLOPs quantifies the computational cost of a single forward pass, it is determined solely by the network architecture and is independent of the IoU evaluation threshold; it is therefore reported once in Table 2 and is not repeated in Tables 4–6, where each model's GFLOPs value is identical to the value listed in Table 2. Figures 9–11 visually present the performance change trend of each model in the range of IoU = 0.6 to 0.9 in the form of attenuation curves. Compared to the baseline, elevating the NMS IoU threshold from 0.6 to 0.9 led to a 2.59% drop in Precision (from 0.849 to 0.827), a 19.31% reduction in Recall (from 0.932 to 0.752), and a 10.67% decrease in mAP (from 0.947 to 0.846). This performance degradation is consistent with the theoretical expectation of increased redundant bounding boxes under a relaxed NMS mechanism. Specifically, a higher threshold allows more overlapping boxes to survive, increasing redundant detections and thus diluting Precision and mAP@50. The diminished Recall further indicates that low-quality duplicate predictions interfered with True Positive determination under relaxed NMS. The baseline model exhibited a more severe attenuation, suggesting a lack of compactness in its initial prediction set, which generated excessive redundancy when NMS constraints were loosened.

**Table 4:** Comparison of mean mAP@50 across different models at an NMS IoU threshold of 0.7.

Model name	Precision	Recall	mAP@50
YOLOv5s	0.842	0.927	0.945
YOLOv5s-BiFPN	0.838	0.930	0.947
YOLOv5s-ASFF	0.833	0.921	0.946
YOLOv5s- Local Contrast	0.844	0.929	0.944
YOLOv5s- CA	0.842	0.937	0.945
YOLOv5s-LCSA	0.833	0.942	0.943
YOLOv5s-LCSA × BiFPN	0.845	0.928	0.945
YOLOv5s-LCSA × ASFF	0.833	0.936	0.945
YOLOv5s-LCSAv2	0.852	0.922	0.947
YOLOv5s-LCSAv2 × BiFPN	0.844	0.928	0.945
YOLOv5s-LCSAv2 × ASFF	0.84	0.94	0.947

**Table 5:** Comparison of mean mAP@50 across different models at an NMS IoU threshold of 0.8.

Model name	Precision	Recall	mAP@50
YOLOv5s	0.872	0.858	0.933
YOLOv5s-BiFPN	0.906	0.836	0.939
YOLOv5s-ASFF	0.873	0.858	0.937
YOLOv5s- Local Contrast	0.882	0.864	0.936
YOLOv5s- CA	0.846	0.891	0.935
YOLOv5s-LCSA	0.881	0.856	0.935
YOLOv5s-LCSA × BiFPN	0.865	0.884	0.937
YOLOv5s-LCSA × ASFF	0.858	0.881	0.936
YOLOv5s-LCSAv2	0.883	0.859	0.937
YOLOv5s-LCSAv2 × BiFPN	0.883	0.851	0.934
YOLOv5s-LCSAv2 × ASFF	0.84	0.91	0.94

**Table 6:** Comparison of mean mAP@50 across different models at an NMS IoU threshold of 0.9.

Model name	Precision	Recall	mAP@50
YOLOv5s	0.827	0.752	0.846
YOLOv5s-BiFPN	0.879	0.761	0.87
YOLOv5s-ASFF	0.826	0.754	0.845
YOLOv5s- Local Contrast	0.856	0.766	0.863
YOLOv5s- CA	0.829	0.754	0.851
YOLOv5s-LCSA	0.860	0.769	0.869
YOLOv5s-LCSA × BiFPN	0.862	0.772	0.87
YOLOv5s-LCSA × ASFF	0.879	0.773	0.878
YOLOv5s-LCSAv2	0.84	0.76	0.849
YOLOv5s-LCSAv2 × BiFPN	0.828	0.766	0.841
YOLOv5s-LCSAv2 × ASFF	0.872	0.756	0.87

The impact of structural improvements on attenuation amplitude varies from model to model. The recall reduction of YOLOv5s-BiFPN is 19.89%, which is similar to Baseline, but the mAP is only attenuated by 8.42%, which is lower than the baseline of 10.67%. The improvement effect of bidirectional feature fusion on positioning stability has initially appeared. The attenuation trend of YOLOv5s-LCSA is gentler, with the recall decrease narrowed to 18.72% and the mAP decrease reduced to 8.04%. The local contrast enhancement mechanism not only strengthens the discriminant features but also contributes to the consistency of frame positioning. The performance of the jointly improved structure further validates the value of module collaboration. The recall and mAP decreases of YOLOv5s-LCSA × BiFPN are 18.05% and 8.13%, respectively, and the degree of attenuation is somewhat convergent compared to the single-module solution. Crucially, YOLOv5s-LCSA × ASFF exhibited the minimal performance degradation as the NMS IoU threshold escalated from 0.6 to 0.9, with the Recall reduction contained at 17.85% and the mAP@50 decline limited to merely 7.19%. Furthermore, at an NMS IoU threshold of 0.9, the mean values of all three metrics for our model either achieved or closely approached the highest levels among all compared networks. This resilient outcome demonstrates that the proposed LCSA × ASFF modification enables the model to generate highly concentrated predicted bounding boxes with superior spatial overlapping for the same lesion. Consequently, the NMS mechanism can effectively eliminate redundancies even under highly permissive conditions, manifesting enhanced robustness to post-processing parameters. The distinct characteristic of highly concentrated predictions indirectly reflects a stronger consistency in the model's localization of lesions, thereby maintaining stable detection performance across diverse clinical deployment scenarios without the necessity for meticulous parameter tuning. Because Tables 2, 4–6 report only the mean over the five folds, inter-arm differences of ≤ 0.01 in mAP (for example, 0.878 vs. 0.870 at IoU = 0.9) cannot be adjudicated without dispersion; across-fold standard deviations, confidence intervals and significance tests were not computed in this study and are required before the ordering of the top-performing arms can be regarded as established rather than within-noise. The non-monotonic precision trend across IoU (rising from 0.6 to 0.8 and then falling at 0.9) is consistent with this sampling variability, since precision at the strictest threshold is estimated from a smaller set of matched detections.

In contrast, the mAP of YOLOv5s-LCSAv2 dropped by 10.63%, and the recall dropped by 19.33%. Both attenuation indicators are close to the Baseline level. The introduction of residual connections failed to bring about the expected improvement in positioning stability, and the gap with the LCSA × ASFF combination is obvious.

The continuous evaluation results of IoU = 0.6 to 0.9 show that the recall of each model shows a downward trend as the threshold increases, but the difference in attenuation amplitude reveals substantial differences in positioning accuracy control between different structures. The mAP and recall decreases of LCSA × ASFF are the smallest among all models. When IoU = 0.9, the three indicators still maintain a high level, and the cumulative effect of positioning errors is effectively suppressed. This result is not an accidental advantage of a single indicator, but the dual characteristics of low attenuation and high absolute value of the combination throughout the continuous threshold interval, which fully reflects the structural robustness.

**Figure 12:**
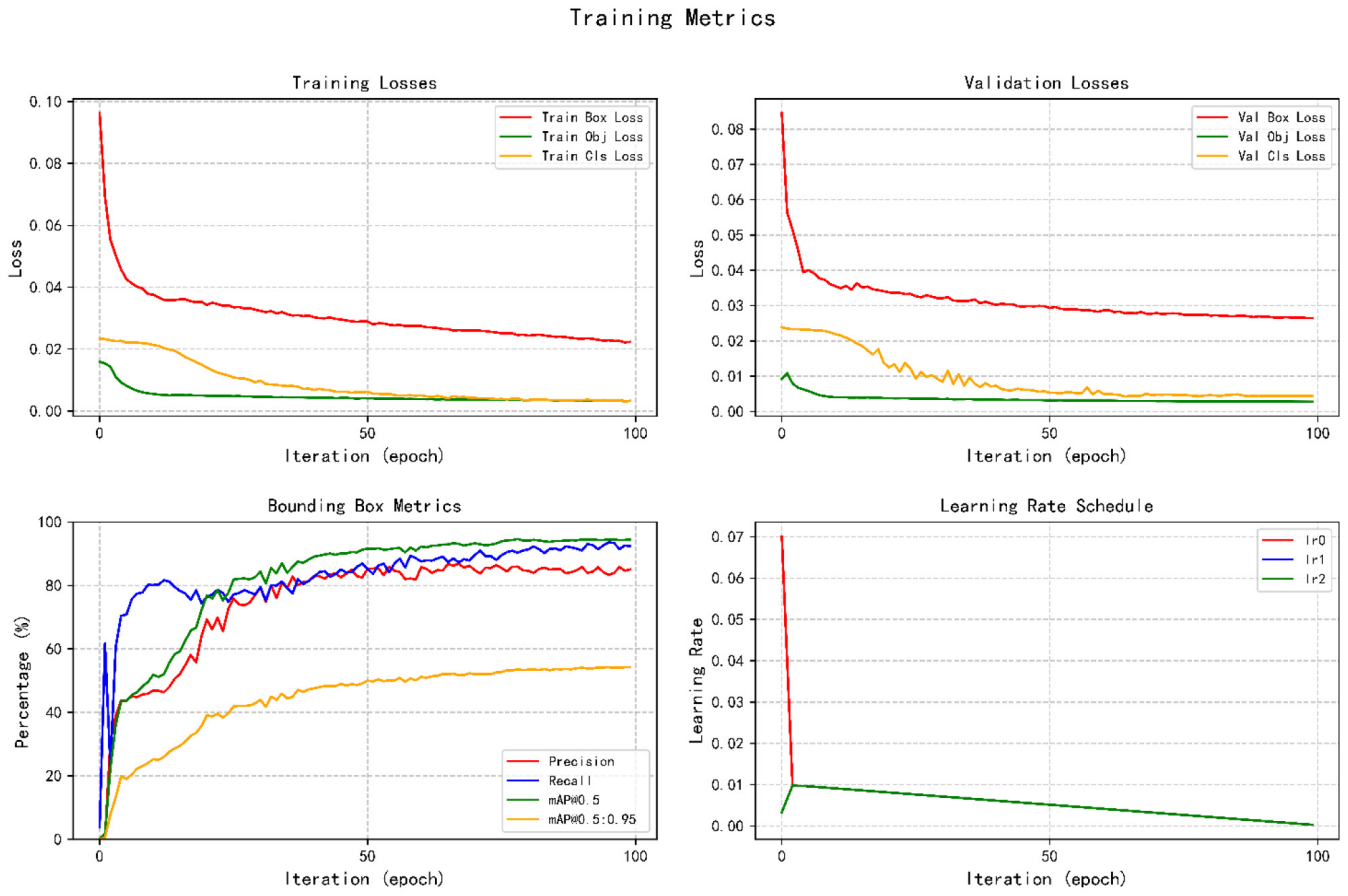
LCSA × ASFF model training curve.

The realization of the above performance is based on the stable convergence of the training process. Figure 12 shows that the training loss and verification loss of YOLOv5s-LCSA × ASFF tend to stabilize after about the 40th epoch, and keep the fluctuation amplitude very small until the end of training. Precision, recall, and mAP continued to climb with the training rounds and finally stabilized. The trends of the loss curves of the training set and the verification set were highly consistent, with no signs of separation, and the training curves of other comparison models also completed convergence. This shows that the introduction of the LCSA × ASFF structure does not interfere with the training dynamics, the model convergence behavior is normal, the risk of overfitting is controllable, the reported evaluation indicators are based on reliable training, and the results are credible.

### 5.5 Test set detection test

In order to further verify the actual effect of the improved module, this study uses the original YOLOv5s model and the optimal weight file improved by introducing LCSA × ASFF to conduct inference evaluation under IoU = 0.9 on the test set.

As shown in Table 7, the precision, recall and mAP@50 of the original YOLOv5s are 0.747, 0.715 and 0.753, respectively, and the corresponding indicators of the improved model are 0.693, 0.753 and 0.788. The precision of the improved model dropped slightly while recall and mAP@50 increased simultaneously. This change is in line with the inherent law of PR trade-off: LCSA enhances the model's sensitivity to low-contrast ground-glass nodules, allowing more weak-signal lesions to be detected. While the recall rate is improved, some false detections are introduced. However, in the AIS and MIA identification scenario, the clinical cost of missed diagnosis is usually higher than misdiagnosis, so this trade-off is reasonable. The overall improvement of mAP@50 shows that the local decrease in precision has been fully compensated by the increase in recall, and the comprehensive detection performance of the improved model is better than the baseline. These test-set values are computed per image; because the 218 images derive from only 27 lesions (about eight correlated slices each), the effective sample size is the number of lesions (*n* = 27) rather than the number of images, so a single misclassified lesion can move the metrics by several points. Lesion-level performance (each lesion counted once) and bootstrap confidence intervals over lesions were not computed here and are needed to quantify this uncertainty; all generalization statements should be read with this limitation in mind.

**Table 7:** Comparison of evaluation index values on the test set before and after improvement.

Model name	*P*	*R*	mAP@50
YOLOv5s	0.747	0.715	0.753
YOLOv5s-LCSA × ASFF	0.693	0.753	0.788

To further characterize computational efficiency on the independent test set, the runtime logs are summarized in Table 8. Compared with the original YOLOv5s model, the LCSA × ASFF model increased the network inference step from 4.8 to 5.5 ms per 640 × 640 image, while the total per-image latency changed from 6.4 to 7.0 ms. Pre-processing and NMS time remained essentially unchanged. Peak memory consumption was not recorded in the exported logs and is therefore not estimated.

**Table 8:** Runtime profile on the independent test set.

Model	Pre-process (ms)	Inference (ms)	NMS (ms)	Total latency (ms/image)
YOLOv5s	0.5	4.8	1.1	6.4
YOLOv5s-LCSA × ASFF	0.5	5.5	1.0	7.0

It should be emphasized that the cross-validation metrics and the independent test-set metrics are not directly comparable, and that the cross-validation values should not be interpreted as estimates of real-world performance. Two factors underlie the apparent gap. First, the two evaluations were obtained at different IoU thresholds: the cross-validation mAP@50 of approximately 0.946 corresponds to IoU = 0.6, whereas the test-set evaluation reported here uses the stricter IoU = 0.9, at which the cross-validation mAP@50 of the improved model is 0.878. Second, and more importantly, the training and validation images were generated by horizontally mirroring approximately 200 original lesions before the five folds were partitioned. Even though rigorous manual screening was performed to remove duplicate lesions, it remains possible that the mirrored counterpart of a given lesion could fall into both the training and validation partitions of the same fold. Such within-fold data leakage would inevitably inflate the validation metrics. The cross-validation results are therefore used only to compare the relative contributions of the different module combinations, whereas the independent test set, comprising 218 original images that never entered training in any form, provides the estimate of the model's generalization to unseen clinical data. The mechanisms underlying this gap are examined further in Section 6.

**Figure 13:**
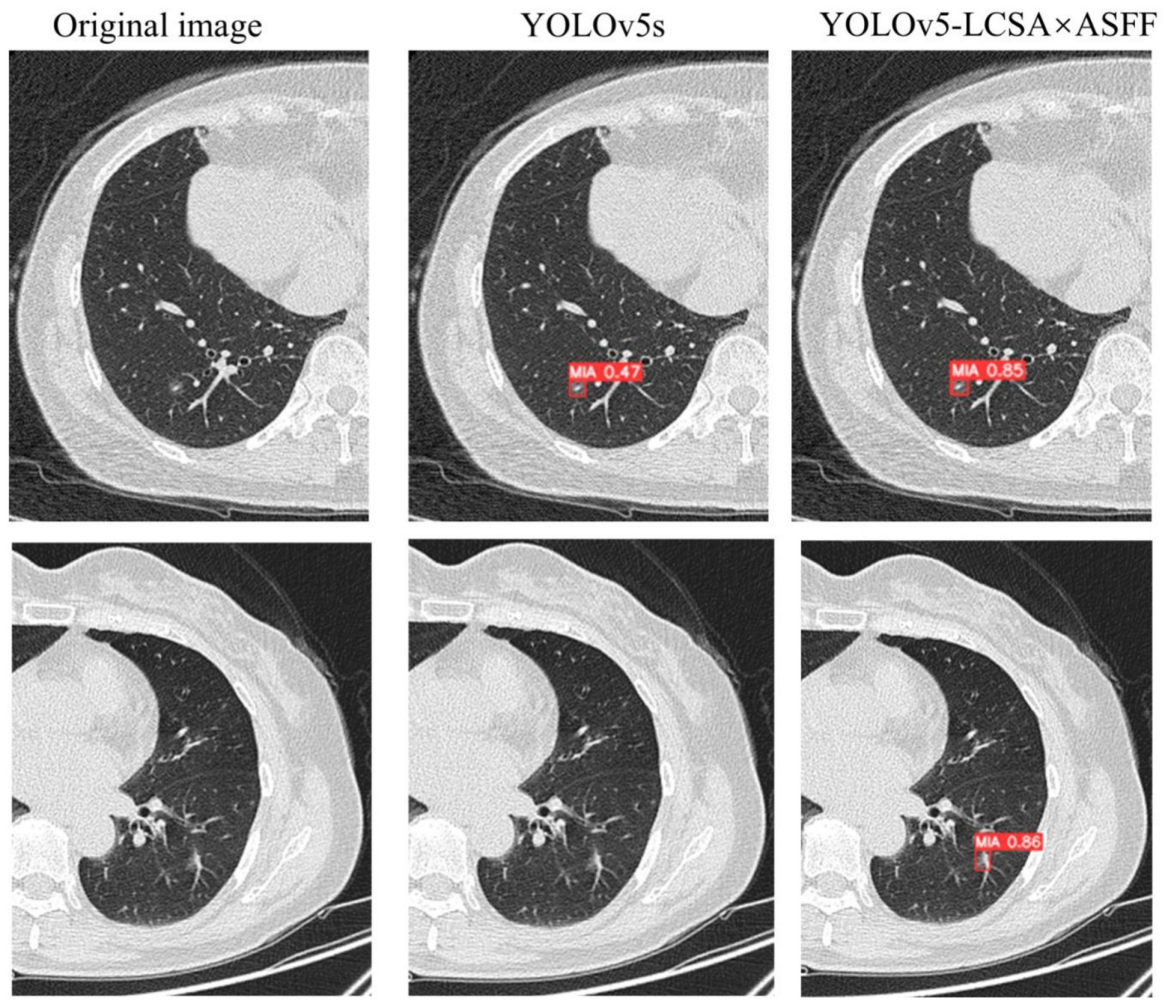
Detection results of lung minimally invasive adenocarcinoma in the model before and after improvement.

**Figure 14:**
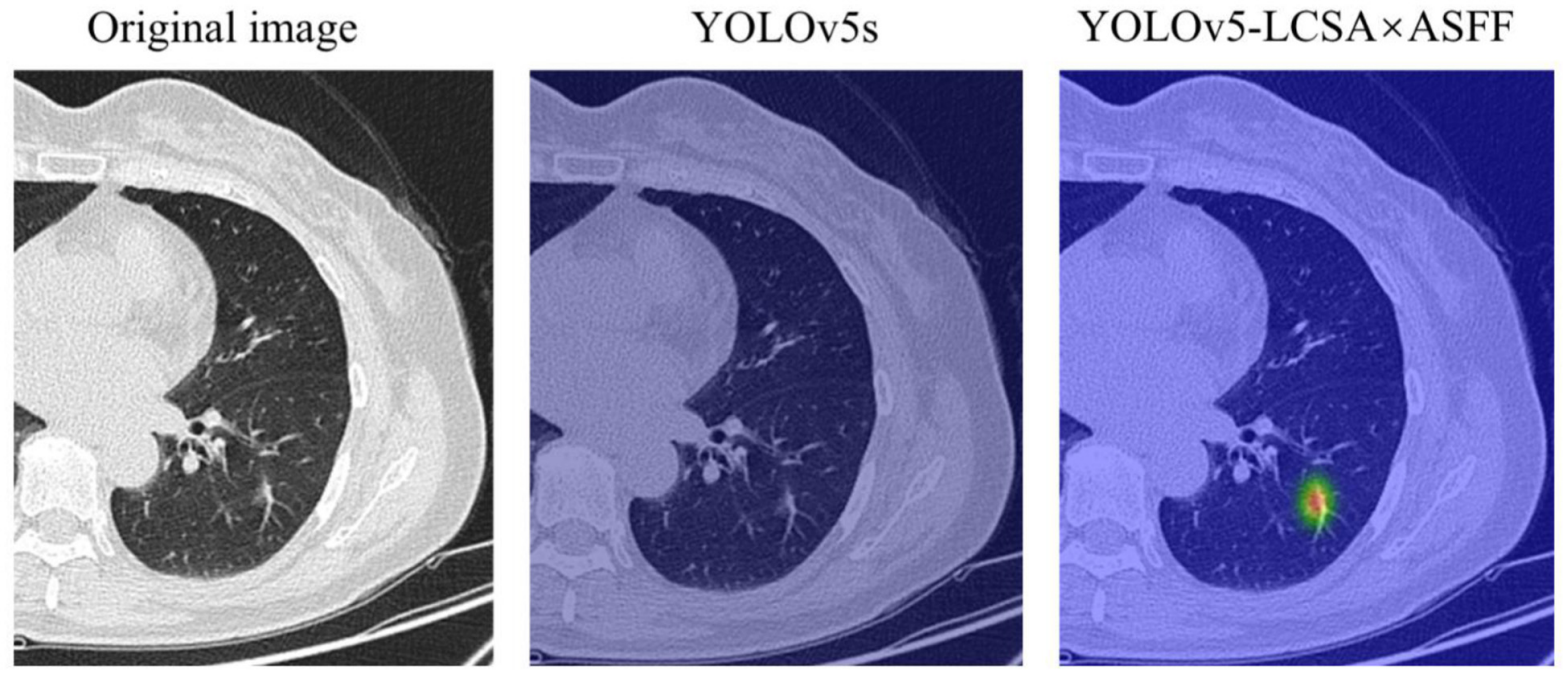
Model heat map activation results before and after improvement.

**Figure 15:**
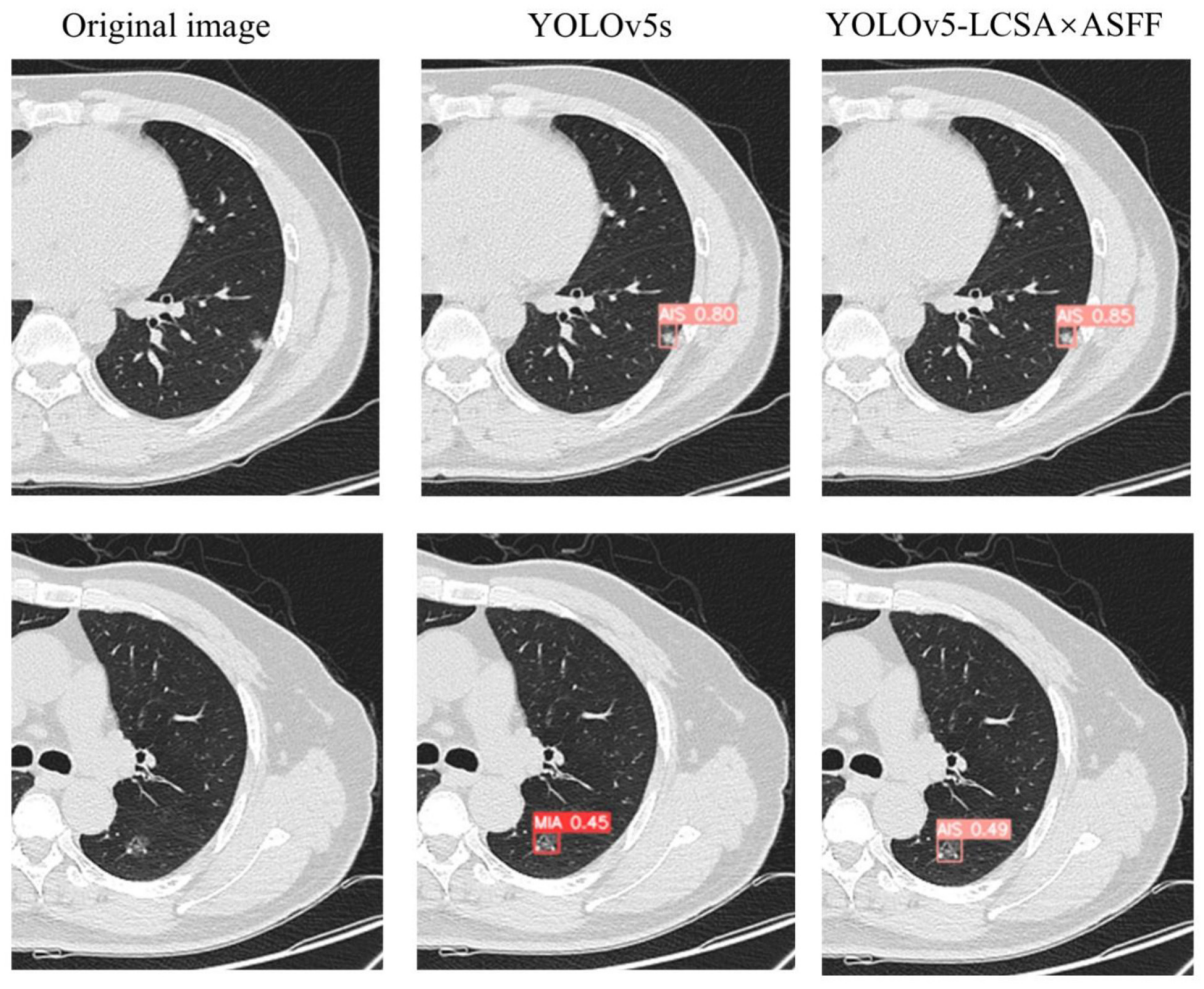
Detection results of pulmonary adenocarcinoma *in situ* in the model before and after improvement.

The visual detection results in Figure 13 show that some samples were missed by the original model, and the improved models all gave correct category predictions. At the same time, in the samples that the original model failed to detect, the improved model successfully completed the detection. The effect of the boundary enhancement and scale adaptive fusion mechanism on suppressing the missed detection rate of small nodules was directly reflected in the actual inference. Figure 14 is the visualization result of the Grad-CAM heat map, which reveals the essential difference between the two models at the feature activation level: the original YOLOv5s heat map shows a uniform low activation state, and no obvious response is produced in the nodule area; the improved model heat map shows that the red high activation core is accurately concentrated in the center of the lesion, the green gradient spreads outward, and the heat distribution is highly consistent with the actual location of the nodule. Figure 15 further shows the correct classification and higher confidence performance of the improved model on samples that were misjudged or missed by the original model.

As shown in Figure 15, the improved model correctly classified samples that were misjudged or missed by the original model with a higher degree of confidence, which intuitively confirmed its substantial improvement in the ability to identify AIS and MIA.

**Figure 16:**
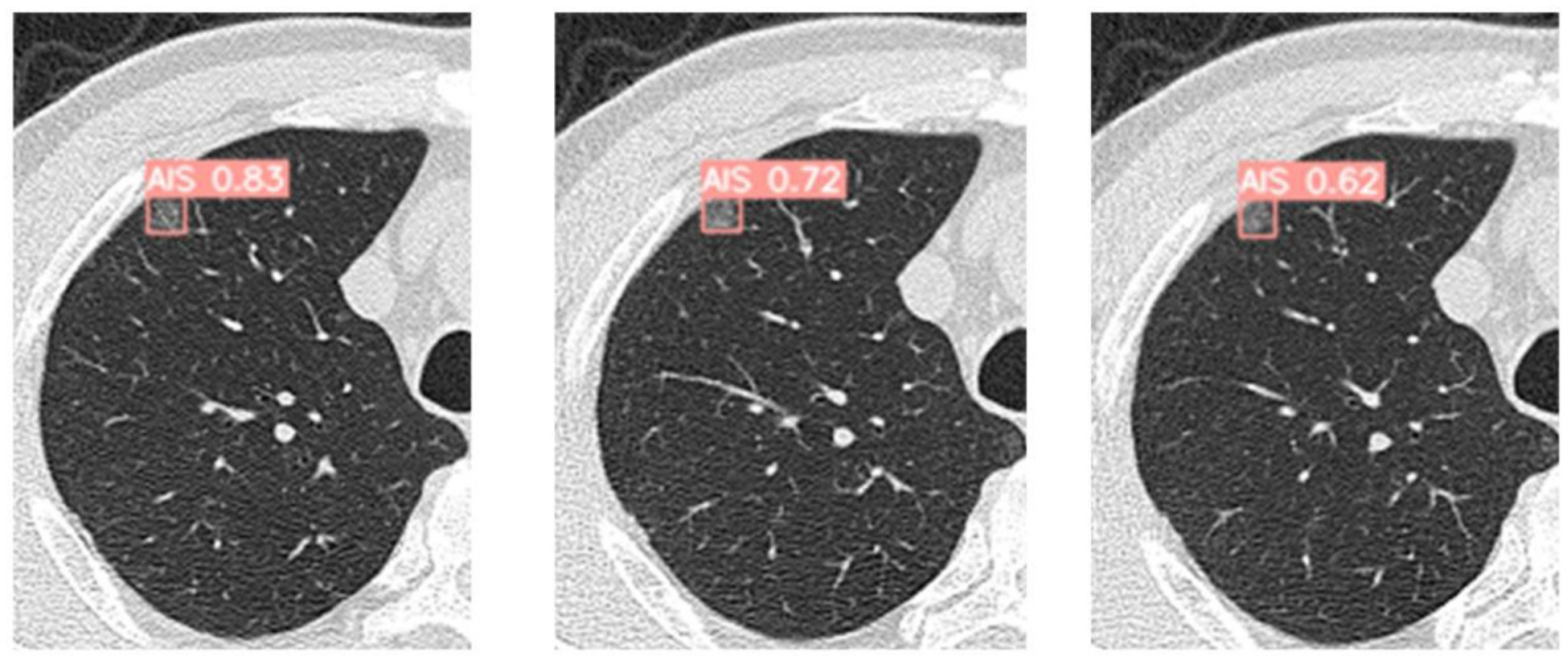
MIA is misjudged as AIS (partial volume effect type).

**Figure 17:**
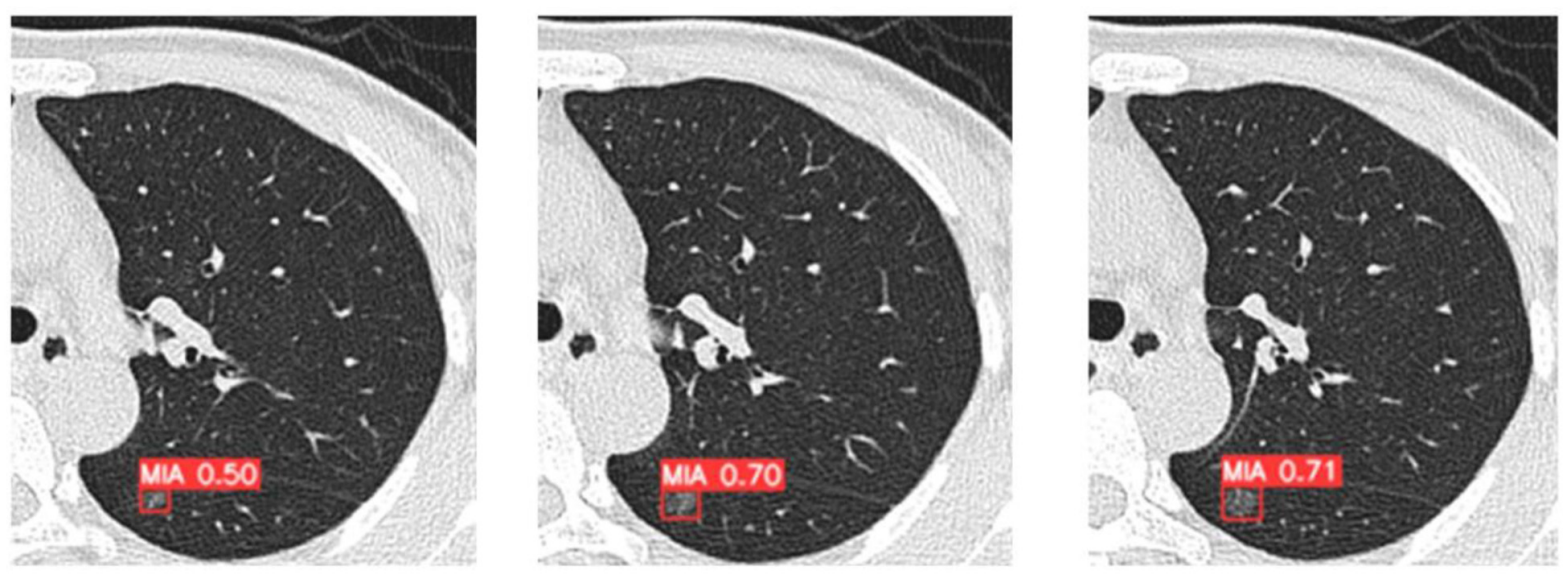
AIS is misdiagnosed as MIA (vascular pseudo-solid type).

As shown in Figures 16, 17, there are two typical misjudgment situations in the false positive samples of the improved model in this study on the independent test set. The first type is partial volume effect type misjudgment, which is confirmed to be MIA by the gold standard of pathology. The model outputs the judgment as AIS at three consecutive levels. The lesion is close to the chest wall and is interfered by the partial volume effect. The second type is the misjudgment of false-solid blood vessels, which is also confirmed to be AIS by the gold standard of pathology. The model outputs judgments at three consecutive levels as MIA. The lesion is adjacent to the blood vessel section, forming a focal false-solid component.

In order to further evaluate the clinical practical value of the improved model, this study invited two doctors with more than 2 years of experience in imaging diagnosis to perform manual interpretation on the same test set as a comparison reference for the automated detection results. Only the classification accuracy of the model was counted on the test set images, and the precision and recall were calculated. The IoU constraints of bounding box positioning were not involved, forming a horizontal comparison. The test set contains a total of 218 images, including 110 MIA images and 108 AIS images, all in PNG format. Two doctors independently completed the classification judgment on the premise that the test set contained two types of lesions. To align with the radiologists' task, the model's precision and recall in Table 9 were computed at the classification level only, without any IoU-based localization constraint; this is why the model's precision in Table 9 (0.832) is higher than its localization-constrained precision in Table 7 (0.693 at IoU = 0.9). It must be stressed, however, that the two assessments are not strictly equivalent: the radiologists performed a pure two-way subtype classification on images already known to contain a lesion, whereas the model first had to detect and localize each lesion before classifying it. The comparison is therefore methodologically asymmetric and should be read as indicative rather than as a like-for-like benchmark.

**Table 9:** Numerical comparison of evaluation indicators between manual and model on the test set.

Model name	*P*	*R*
Human interpretation	0.684	0.669
YOLOv5s-LCSA × ASFF	0.832	0.753

As shown in Table 9, the manual interpretation results show a certain proportion of misclassifications, and there are still certain difficulties in distinguishing the two types of lesions. At the class level for the improved model, MIA precision and recall were 0.907 and 0.618, respectively, while AIS precision and recall were 0.756 and 0.889, respectively; the macro-averaged precision and recall were therefore 0.832 and 0.753, as reported in Table 9. In this calculation, no-detection outputs were counted as false negatives for recall but were not added to the false-positive denominator for precision. The average precision of the improved model on the test set was 0.832, and the average recall rate was 0.753, which was numerically higher than the two readers' values; as detailed below, however, this is not a like-for-like comparison. The results initially showed that it has certain application potential in assisting preliminary screening and classification, but its clinical practicality still needs to be further verified by multi-center data. Accordingly, the observation that the model's classification-level precision and recall exceed those of the two readers should be interpreted with caution. The two readers classified isolated axial PNG slices without access to the full volumetric scan or the surrounding clinical context, the evaluation rests on a small sample (27 lesions, 218 images), and the model and the readers were assessed under different conditions (detection-plus-classification scored at the classification level vs. classification alone). This comparison therefore serves only as a preliminary indication of potential utility and does not establish that the model outperforms experienced radiologists in routine clinical practice.

For paired significance testing, the original and improved model outputs were matched by image filename and analyzed using McNemar's exact test (Table 10). Consistent with the precision/recall definitions, no-detection outputs reduce recall but are not counted as false-positive predictions; for McNemar's binary paired analysis they were coded as incorrect. To avoid overstating performance, mixed-class outputs containing both AIS and MIA were also treated as non-completely correct. The discordant pairs were balanced (23 original-incorrect/improved-correct images vs. 18 original-correct/improved-incorrect images), and the difference did not reach statistical significance (*p* = 0.533). Therefore, the test-set change should be interpreted as a numerical trend requiring larger lesion-level and external validation rather than as a statistically confirmed superiority claim.

**Table 10:** Paired McNemar test comparing original and improved models on the same 218 test images.

Analysis definition	Original incorrect/improved correct (b)	Original correct/improved incorrect (c)	Exact *p-value*
Strict image-level correctness; no-detection and mixed-class outputs coded as incorrect	23	18	0.533

## 6 Discussion

This study aims at the problem of identifying pulmonary adenocarcinoma *in situ* (AIS) and minimally invasive adenocarcinoma (MIA) with low-contrast ground-glass nodules, blurred boundaries, and subtle features on CT images. This study proposes a deep learning detection model based on improved YOLOv5s. The core lies in the introduction of a collaborative mechanism between the local contrast-spatial attention module (LCSA) and the adaptive spatial feature fusion (ASFF). Continuous evaluation results under high IoU conditions show that the improved model maintains a good balance between precision and recall. Test set analysis (Figures 13–17) shows that the LCSA × ASFF module significantly improves the detection rate of micro-nodules and low-contrast lesions while maintaining boundary positioning stability. Compared with the baseline model, the improved model can maintain stable box regression performance under high thresholds, and its spatial positioning accuracy has direct clinical significance in determining the boundary between AIS and MIA.

The decline of mAP@50 as the NMS IoU threshold escalates represents a systematic phenomenon inherent to the post-processing phase of object detection. Under low IoU conditions, when the prediction frame roughly covers the target area, it can be judged as a correct detection, and recall is therefore maintained at a high level; when the threshold rises to 0.8 or even 0.9, the boundary deviation of the same magnitude is enough to directly cause detection failure, and the penalty slope of the IoU function for positioning error is non-linearly amplified as the threshold increases. An increased NMS threshold implies a higher tolerance for overlapping candidate boxes, allowing more redundant detections to survive. Consequently, the volume of False Positives in the model output escalates, thereby diluting both Precision and mAP@50. The variance in degradation magnitudes across different models reveals a fundamental disparity in prediction box quality: networks yielding more concentrated bounding boxes with minimal redundancy are less susceptible to relaxed NMS constraints. Specifically, the local contrast sensitivity attention (LCSA) branch enhances gradient responses along lesion boundaries, forcing multiple predictions for the same lesion to highly converge spatially, which subsequently elevates the inter-box overlap. Synergistically, the adaptive spatial feature fusion (ASFF) further guarantees consistent feature representation across multiple scales, reducing scattered predictions induced by scale mismatch. The orchestration of these two components ensures that the LCSA × ASFF combination delivers higher-quality prediction boxes, maintaining stable detection performance under shifting NMS parameters. This post-processing robustness holds direct practical utility for clinical deployment so that the regression head can accurately fit the target contour coordinates (49), the spatial attention weight distribution must be accurately focused on the target area to prevent background interference from causing feature response drift (50). LCSA's dual-branch structure is a directional solution designed for these two sources of failure.

The mechanism of the local contrast enhancement branch can be understood from the response characteristics of the convolution operation. Depthwise separable convolution performs independent local filtering on each channel feature map, and enhances areas with drastic grayscale changes by comparing the differences between central pixels and surrounding pixels (51). Although multiple stacking of standard convolutions can extract high-level semantics, it also brings the side effect of gradual smoothing of the feature map, causing the originally weak boundary gradient signal of the lesion to be gradually diluted in the process. The intervention of the local contrast enhancement branch is precisely to find these smoothed gradient information boundaries to form a clear intensity stratification between the edge of the lesion and the internal area. Therefore, the regression head obtains a more reliable positioning reference instead of relying on a fuzzy response area. The edge fitting error is narrowed under high IoU threshold. When the network can judge the boundary, accurate positioning is possible.

The coordinate attention branch solves the localization problem from another dimension. CoordAtt performs one-dimensional pooling along the horizontal and vertical directions, encodes spatial position information into directional attention weights, and performs spatial calibration on the original feature map with element-wise multiplication. The SE module only reweights the channel dimension, and the spatial position resolution is therefore lost; CoordAtt retains accurate object localization capabilities at the row and column coordinate level (52). Small nodules are often surrounded by complex lung textures. These non-target areas are prone to false high activations on the feature map, causing the network to mistakenly think that there is something here, and the center point of the prediction frame is shifted accordingly. The role of CoordAtt is that it constrains the activation weights along the horizontal and vertical directions, and filters out false responses that deviate from the focus queue. The impact of background interference on positioning is effectively suppressed, and the attenuation trend of the recall rate in the mid-to-high IoU range is therefore flattened.

The synergistic effect of the two branches results from the complementary properties of the spatial activation distribution. The output of LocalContrast takes the target boundary as the response peak, and the attention weight of CoordAtt takes the target center area as the high value. After additive fusion, the target area obtains both high boundary gradient response and high spatial attention weight, and both signals in the background area are in a low activation state. This complementary superposition allows the model to benefit from the dual improvement of edge coordinate fitting accuracy and center positioning stability under high IoU thresholds, forming the core mechanism for LCSA to stabilize high IoU evaluation indicators.

The choice of LCSA insertion position has a substantial impact on the functioning of the above mechanisms. The P3 layer has the highest spatial resolution, and the fine-grained boundary information of small targets can be completely preserved here (53). After the Concat fusion operation, the high-level semantic features from the upsampling path and the low-level spatial details output by Backbone P3 have been spliced in the channel dimension. The C3 module then integrates the cross-channel features, so that the feature map before entering the LCSA carries the dual expression of semantic context and spatial details. LCSA therefore operates on a feature map with the highest information density and no spatial resolution loss due to downsampling. This location choice allows the advantages of the two branches to be fully released. LocalContrast can capture local gradients from pixel-level texture changes, and CoordAtt can also rely on complete spatial resolution to achieve precise coordinate positioning, allowing the model to perceive the shape of the lesion and lock the specific location of the lesion.

After LCSA and ASFF are jointly deployed, the performance distribution of the model's high IoU range shows more balanced characteristics. This phenomenon needs to be understood from the failure modes targeted by each of the two modules. It enhances local contrast on the high-resolution layer P3 and significantly improves the ability of single-layer features to describe the boundaries of lesions. However, the field of view of LCSA is limited to a single level, and its design itself does not address the cross-scale information matching problem caused by changes in nodule size. ASFF just fills this gap. It dynamically evaluates the contribution of features at different levels through learnable fusion weights (54), when the nodule size is small, the detailed information of the P3 layer is given higher weight. When nodules require richer context, the semantic features of P4 and P5 layers are moderately introduced. The issue of scale adaptation has been responded to by ASFF.

All in all, the division of labor between LCSA and ASFF can be summarized as directional intervention in two mutually independent error dimensions: the former acts within the feature layer to improve the quality of boundary expression; the latter spans between feature layers to ensure the stability of scale matching. The source of the prediction box deviation under high IoU conditions corresponds to these two dimensions. Unclear boundary gradients lead to edge coordinate fitting deviations, and unstable scale matching leads to scale errors in the overall box. The two types of failure modes are independent of each other, and a single module can only target one of them. Joint deployment will block both loopholes at the same time, ensuring that the model's positioning accuracy for nodules of different scales and contrasts is guaranteed.

In the experimental results, the performance curve and heat map visualization results of the LCSA × ASFF combination exactly confirm this mechanism analysis. Within the range of IoU = 0.6 to 0.9, the performance curve of this combination is not only higher than other solutions overall, but also has the gentlest downward trend. The improvement in the overall level is due to the superimposed benefits of double error suppression, and the smooth attenuation trend is due to the complementary coverage of different target conditions by the two mechanisms. No matter how the target scale changes and the contrast fluctuates, both types of error sources have corresponding mechanisms to suppress them. The performance curve therefore maintains stronger stability under strict positioning standards. And it can be observed on the heat map that the model accurately locates and judges the core and boundary of the lesion.

The reason for the decline in the synergy effect after combining LCSAv2 with ASFF and BiFPN is not due to the performance defects of LCSAv2 itself, but to the functional overlap between its residual connection design and fusion module, which makes it difficult to effectively complement each other when deployed in series. This mechanistic account of the LCSAv2 residual is a *post-hoc* interpretation: the residual connection was not ablated in isolation and no dispersion estimates are available, so, given the small inter-arm differences, the apparent disadvantage of LCSAv2 may partly reflect run-to-run variation and should be treated as a hypothesis rather than an established result.

The original intention of introducing residual connections in LCSAv2 is to retain the integrity of the original semantic information and prevent excessive local contrast enhancement from causing feature distortion. Under single module conditions, this design does give the module certain stability advantages. However, the main image characteristics of AIS and MIA lesions are low contrast and blurred boundaries, and the target discrimination signal is already in a weak activation state. The introduction of residual connections allows the low-frequency components of the background to be dually preserved through two paths. The residuals of the enhanced branches and the direct transmission of the skip connections work at the same time. The enhanced reconstruction ability of the local contrast branch for weak signals is therefore subject to rigid constraints, making it difficult to give full play to the design advantages of low-contrast targets.

This structural contradiction is further amplified when deployed jointly with ASFF or BiFPN. ASFF uses position-adaptive fusion weights to suppress conflicts between scales and strengthen complementary information during cross-layer integration, objectively maintaining the discriminative representation of features at each scale (55), BiFPN uses learnable bidirectional fusion coefficients to adaptively balance the contributions of each layer in multiple top-down and bottom-up information round-trips, and the core semantics and fine-grained details of multi-scale features are retained (56). The function of retaining original information undertaken by LCSAv2 residual connection highly overlaps with the above-mentioned fusion mechanism, and the original low-frequency background components are therefore double protected in the network. This kind of functional redundancy compresses the operating space for the fusion module to dynamically adjust the weight of discriminative features, thereby weakening the responsiveness of the scale matching problem, and the overall gain after the two modules are superimposed is lower than expected.

The effectiveness of the combination of LCSA and ASFF stems precisely from the fact that there is no functional overlap between the two. LCSA does not contain residual connections, has a large transformation amplitude on input features, and the output is a fully reconstructed highly discriminative feature representation. Based on this, ASFF uses an adaptive weight mechanism to dynamically select the optimal feature layer combination for the current target. The two modules respectively address the two independent issues of intra-layer feature quality and inter-layer scale consistency. The functional boundaries are clear, the information flow path is smooth, and collaborative gains under strict positioning conditions with high IoU are achieved.

The experimental results of LCSAv2 provide an enlightening comparison from the opposite side. The introduction of residual connections failed to bring expected benefits, but instead weakened the module's reconstruction ability in low-contrast and weak-signal scenarios, revealing design principles worthy of attention. For small object detection tasks with low contrast and blurred boundaries, feature reconstruction is often more practical than retaining original features. The local contrast enhancement branch extracts submerged boundary signals from the background, while the original information path introduced by the residual connection weakens this reconstruction process. Therefore, in the design of attention modules for low-contrast lesions such as AIS/MIA, the signal enhancement strategy is more suitable than the residual strategy that retains the original semantics. This has reference significance for the subsequent attention module design for small object detection in medical images.

This study observed a significant gap between the performance index of the independent test set and the mean value of the five-fold cross-validation. The primary reason is the impact of the data augmentation strategy on the cross-validation results of this study. The training and validation sets were horizontally mirrored amplified from about 200 original lesions, and then divided into five-fold datasets. Furthermore, the data augmentation in this study was performed prior to the partitioning of the training and validation sets. Although rigorous tracking of lesion sources was employed to mitigate the risk of cross-dataset distribution of homologous images, the substantial volume of augmented samples and the high similarity among certain images preclude the absolute exclusion of potential correlation among a fraction of these samples. Consequently, the performance metrics obtained during the cross-validation phase might be relatively optimistic and may not fully reflect the generalization capability of the model in real-world clinical settings. The independent test set consists of 218 original images that have not participated in any training process, and its evaluation results are closer to the actual performance of the model on real clinical data. Secondly, the test set was extracted from 27 lesions, and the sample size is limited. The distribution of extreme cases has a greater impact on the overall index, and the statistical stability of the performance estimate is limited. The cross-validation index is used to evaluate the relative contribution and structural optimization effect of each improved module, and is used to compare the performance differences between different module combinations. The test set is used to evaluate the actual generalization ability of the model on unenhanced original clinical images, reflecting the application potential of the improved model in real diagnostic scenarios. However, due to the limitation of the test set size, it cannot fully represent the overall generalization ability of the model, and it needs to be expanded to a larger external test set for confirmation.

This study selected two groups of typical false positive cases from the independent test set for analysis to reveal which types of lesions still have a tendency to systematically misjudge under the current model framework.

The first type of false positive: MIA is misjudged as AIS. As shown in Figure 16 above, the lesion is located in the anterior segment of the right upper lobe of the lung near the chest wall, and the pathological diagnosis is MIA. However, the model outputs AIS predictions at three consecutive levels, and the confidence fluctuates. The anatomical location of the lesion near the chest wall causes the partial volume effect of soft tissue to intervene, which reduces the overall average density of the lesion. The actual tiny solid components are obscured by the diffuse low-density background, making it difficult to form a significant contrast on the density gradient. At the same time, the lesion shape is flat and diffuse, lacking the round-like outline common in MIA, and the weight of the morphological features of the model is therefore shifted toward pure ground glass AIS. The confidence level oscillates between the three layers instead of changing monotonically, which reflects that the model is in a continuous swing state at the decision boundary between AIS and MIA on this lesion, which is a typical manifestation of uncertain signals.

The second type of false positive: AIS is misjudged as MIA. As shown in Figure 17 above, the lesion was located in the mediastinal area near the mediastinum of the lower lobe of the right lung. The overall density was mainly ground glass, and the pathological diagnosis was AIS. The model outputs MIA predictions at three consecutive levels, and the confidence level shows a monotonically increasing trend. Careful observation of the area within the marked box shows that there are punctate high-density areas in the lesion. Combined with the anatomical location of the lesion near the mediastinum, the high-density area is judged to be the blood vessel cross section produced when the branches of the pulmonary veins travel along the plane direction, that is, the “pseudo-solid blood vessel” phenomenon. The density feature of the blood vessel cross section on the axial image highly overlaps with the solid component in the definition of MIA in the feature space, and there is a slight increase in background density in the surrounding lung tissue, which further provides the high-density point with an image background similar to the “ground-glass halo sign,” causing the model to incorrectly decode the combination of pseudo-solid blood vessels and low-contrast ground-glass background as the image pattern of MIA. The characteristic that the confidence level increases monotonically with the layer also confirms the model's continuous error-enhanced recognition process of “pseudo-solid components” along the direction of blood vessels.

The above two types of false positive cases reveal the systematic misjudgment rules of the model in specific anatomical locations. The proximal mediastinal area is rich in blood vessels, and the blood vessel cross section is easily misidentified as a solid component, which constitutes the main reason for misjudgment of AIS to MIA. The partial volume effect in the near chest wall area is significant, and the true solid component is easily diluted by density, which is the main reason for misdiagnosis of MIA to AIS. Both types of errors point to the same deep defect. The model's perception of the solid component relies on the absolute magnitude of the local density peak, and lacks the ability to model the relative density gradient between it and the surrounding ground glass halo.

These two types of false positive cases jointly reveal that the model's judgment of solid components relies too much on local density peaks and lacks the ability to perceive three-dimensional spatial continuity and relative density gradients. Future research can explore the density contrast relationship between high-density points inside the lesion and its surrounding ground-glass background in the feature extraction stage, and track the spatial continuity of suspected solid structures along the layer direction, thereby reducing systematic misjudgments caused by pseudo-solid vascularity and partial volume effects at the source.

This study has several limitations. (1) The dataset was derived from a single centre and the number of underlying lesions in the independent test set was limited. The independent test set was kept completely separate from augmentation and training, so it is useful for examining generalization on unaugmented images; however, it remains an internal test set, and the 218 images were correlated slices from only 27 lesions. External multicentre validation is therefore necessary, but it is difficult to implement immediately because cross-hospital CT data collection requires separate ethics approval, data-use agreements, patient privacy review, and unified annotation/quality-control procedures. (2) The scope of the comparative experiment is limited to the convolutional neural network framework, and the large model architecture represented by Transformer is not included in the horizontal comparison. This model has potential advantages in long-range dependency modeling and global context awareness (57), its actual performance in the small nodule detection task is still unclear. Subsequent research can conduct a more systematic comparative analysis in this direction to further explore the suitability of different models in this task. (3) The insertion position of the attention and fusion modules was chosen on the basis of architectural reasoning rather than an exhaustive search: LCSA, LCSAv2, and ASFF were embedded at the P3 detection head because P3 retains the highest spatial resolution and therefore incurs the least downsampling-induced information loss for small targets. A systematic ablation of alternative insertion positions (for example, LCSA at the P4 or P5 detection head) was not conducted in the present study and is left to future work. (4) Because the AIS/MIA class labels were taken from the postoperative pathological gold standard and the bounding boxes were produced by consensus rather than by independent duplicate annotation, a quantitative inter-rater agreement metric (such as Cohen's kappa) was not computed; future work will incorporate independent double annotation to report annotation reliability.

## 7 Conclusion

Using AIS and MIA differential diagnosis as the application scenario, this study constructed and validated an improved YOLOv5 deep learning model for the automated detection and classification of small pulmonary nodules. The core improvements comprised synergistic deployment of the LCSA local contrast-spatial attention module and the ASFF multiscale adaptive feature fusion mechanism, enabling precise localization and stable classification of small low-contrast lesions. Continuous evaluation under high IoU constraints demonstrates that the improved model maintains stable Precision, Recall, and mAP values. Furthermore, it exhibits the minimal performance degradation during sequential adjustments of the NMS IoU threshold from 0.6 to 0.9, indicating superior bounding box prediction quality and enhanced robustness toward post-processing parameters. Overall, the improved YOLOv5 model demonstrates encouraging accuracy, sensitivity to small nodules, and boundary stability in AI-assisted diagnosis of AIS and MIA, providing a candidate technical approach to assist screening for adenocarcinoma *in situ* and minimally invasive adenocarcinoma.

## Data Availability

The datasets presented in this article are not readily available because this could lead to data leakage, thereby compromising patients' privacy. Requests to access the datasets should be directed to Zhipeng Sun, 125396285@qq.com.

## Appendix A. Pseudocode of the LCSA and LCSAv2 modules

**Table A1:** LCSA_Block forward pass.

Input: Feature map X ε R^∧^(N × C × H × W) Output: Enhanced feature map Out ε R^∧^(N × C × H × W) 1: lc ← DepthwiseConv(X, kernel=3, groups=C) 2: lc ← BatchNorm(lc) 3: x_h ← HorizontalAvgPool(X) //R^∧^(N × C × H × 1) 4: x_w ← VerticalAvgPool(X) //R^∧^(N × C × 1 × W) 5: f ← Concat(x_h, x_w, dim=H) //R^∧^(N × C × 1 × (H+W)) 6: f ← SiLU(BatchNorm(Conv1 × 1(f))) 7: x_h, x_w ← Split(f, [H, W], dim=H) 8: A_H ← Sigmoid(Conv1 × 1(x_h)) //R^∧^(N × C × H × 1) 9: A_W ← Sigmoid(Conv1 × 1(x_w)) //R^∧^(N × C × 1 × W) 10: sa ← X ⊗ A_H ⊗ A_W 11: out ← lc + sa 12: out ← SiLU(BatchNorm(Conv1 × 1(out))) 13: return out

**Table A2:** LCSA_Block_v2 forward pass.

Input: Feature map X ε R^∧^(N × C × H × W) Output: Enhanced feature map Out ε R^∧^(N × C × H × W) 1: lc ← DepthwiseConv(X, kernel=3, groups=C) 2: lc ← BatchNorm(lc) 3: lc ← SiLU(lc) // 4: x_h ← HorizontalAvgPool(X) //R^∧^(N × C × H × 1) 5: x_w ← VerticalAvgPool(X) //R^∧^(N × C × 1 × W) 6: f ← Concat(x_h, x_w, dim=H) //R^∧^(N × C × 1 × (H+W)) 7: f ← SiLU(BatchNorm(Conv1 × 1(f))) 8: x_h, x_w ← Split(f, [H, W], dim=H) 9: A_H ← Sigmoid(Conv1 × 1(x_h)) //R^∧^(N × C × H × 1) 10: A_W ← Sigmoid(Conv1 × 1(x_w)) //R^∧^(N × C × 1 × W) 11: sa ← X ⊗ A_H ⊗ A_W 12: out ← lc + sa 13: out ← SiLU(BatchNorm(Conv1 × 1(out))) 14: out ← out + X // 15: return out
